# Distinct Cause of Death Profiles of Hospitalized Non-alcoholic Fatty Liver Disease: A 10 Years' Cross-Sectional Multicenter Study in China

**DOI:** 10.3389/fmed.2020.584396

**Published:** 2021-01-12

**Authors:** Yansong Lin, Xiaorong Gong, Xin Li, Congxiang Shao, Tingfeng Wu, Minrui Li, Fuxi Li, Qianqian Ma, Junzhao Ye, Bihui Zhong

**Affiliations:** ^1^Department of Gastroenterology, The First Affiliated Hospital, Sun Yat-sen University, Guangzhou, China; ^2^Department of Gastroenterology, First Affiliated Hospital of Guangzhou Medical University, Guangzhou, China; ^3^Department of Gastroenterology, Affiliated Dongguan People's Hospital, Southern Medical University (Dongguan People's Hospital), Dongguan, China

**Keywords:** cause of death, non-alcoholic fatty liver disease, steatosis degree, multiorgan failure, real world study

## Abstract

**Background:** The clinical burden and natural history of non-alcoholic fatty liver disease (NAFLD) vary globally. We aimed to investigate NAFLD-related mortality profiles in hospitalized patients in southern China.

**Methods:** A multicenter retrospective investigation with a 10-year study period (2009–2018) analyzed 10,071 deaths during hospitalization (NAFLD: 2,015; other liver diseases: 1,140; without liver diseases: 6,916) was performed using a multiple cause of death analysis. Medical histories and biochemistry and imaging findings were extracted from the electronic medical record system. The underlying causes of death were classified by 10th Revision of the International Classification of Diseases (ICD-10) codes.

**Results:** The distribution of death causes in patients with NAFLD has stabilized over time, with cardio- and cerebral vascular disease (CVD) ranked first (35.6%), followed by extrahepatic malignancies (22.6%), infection (11.0%), kidney disease (7.5%), liver-related diseases (5.2%), respiratory diseases (3.9%), digestive diseases (3.5%), endocrine diseases (3.5%), and other diseases (7.2%). NAFLD patients had more deaths attributable to CVD, extrahepatic malignancies, liver-related diseases (all *P* < 0.001) and multiorgan failure than the deceased controls. The severity of steatosis was independently associated with these relationships (liver-related diseases: OR = 1.37, 95% CI: 1.20–1.59, cardio- and cerebrovascular diseases: OR = 1.23, 95% CI: 1.19–1.31, infectious diseases: OR = 1.14, 95% CI: 1.04–1.26, and renal diseases: OR = 1.21, 95% CI: 1.02–1.47, all *P* < 0.05) after adjustment for sex, body mass index (BMI), fasting blood glucose, low-density lipoprotein cholesterol, uric acid, metabolic syndromes and fibrosis index based on the 4 factors.

**Conclusion** : NAFLD patients had higher proportions of death due to underlying CVD and liver-related diseases than the general population in China; these proportions positively correlated with steatosis degree.

## Introduction

Non-alcoholic fatty liver disease (NAFLD) has become the primary cause of chronic liver diseases in the last decade, affecting over one-fourth of the population worldwide (1). As one of the major drivers of hepatic mortality in developed countries (1–3), there is accumulating evidence that NAFLD is also responsible for extrahepatic system diseases. Epidemiologic data demonstrated that NAFLD was not only associated with a collection of metabolic syndrome components but also promoted the development of type 2 diabetes, cardiovascular disease (CVD) (4–6), osteoporosis, chronic kidney disease (7), chronic obstructive pulmonary disease (8, 9), pneumonia (10), sleep apnea, urinary tract infection (11), gallstone disease (12), polycystic ovary syndrome, and malignant neoplasms (13) independently or *via* shared metabolic comorbidities (3).

Although NAFLD is strongly associated with and contributes to the clinical burden of such specific complications, whether it contributes to mortality due to distinct liver-related and extrahepatic diseases remains unknown (14–16). A recent meta-analysis reported that NAFLD increased overall mortality by 57% compared with the general population; mortality was mainly attributable to liver disease and CVD (17). Moreover, a recent meta-analysis including 25,837 patients from a six-cohort study demonstrated that the risk of clinical cardiovascular events was significantly higher in patients with NAFLD than in those without NAFLD (RR: 1.77; 95% CI: 1.26–2.48, *P* < 0.001) (18). However, in a more recent longitudinal study with a 10-year follow-up in southern Asia, a lack of an association between NAFLD and cardiovascular mortality was reported (19). As most reports to date have utilized Gaussians, whether disparities in causes of death exist across ethnicities or regions remains unclear.

Epidemiological studies have indicated that the prevalence of NAFLD continues to increase in Asia (20), with the highest incidence predicted in China between 2016 and 2030 (21). The aforementioned studies suggested that Asian patients with NAFLD exhibited metabolic differences compared to Caucasians. Lean Asian people are more prone to metabolically obesity and insulin resistance than Europeans because they accumulate more adipose fat at an equivalent body mass. Moreover, genetic backgrounds, dietary habits, and lifestyles differ greatly between Western countries and Asia. The specific pattern of disease progression in Asian NAFLD populations has been limited by small sample sizes and short follow-up periods. Studies on the distribution of death causes in the disease course of Asian patients with NAFLD are scarce in Asia.

The aim of this study was to examine whether NAFLD was associated with a distinct spectrum of underlying causes of death in Chinese adults and to determine whether the degree of steatosis differentially influenced this relationship. Moreover, we also identified the clinical characteristics of patients with different causes of death in the setting of NAFLD.

## Materials and Methods

### Ethics

The study involving human participants were reviewed and approved by Clinical Research Ethics Committee of the First Affiliated Hospital of Sun Yat-sen University, the First Affiliated Hospital of Guangzhou Medical University, and the Affiliated Dongguan People's Hospital of Southern Medical University and written informed consent was obtained from all patients.

### Study Population and Design

This was a retrospective survey utilizing a medical record review of consecutive hospitalization deaths from three tertiary university-affiliated medical centers in southern China (The First Affiliated Hospital of Sun Yat-sen University, The First Affiliated Hospital of Guangzhou Medical University, and the Affiliated Dongguan People's Hospital of Southern Medical University) from January 1, 2009, to December 31, 2018. The study was approved by the institutional and regional medical ethics committees. The inclusion criterion was patients aged over 18 years who underwent an abdominal ultrasonography (US) examination. Diagnosis of NAFLD were defined as those who met at least one of the three ultrasound criteria for fatty liver, and did not have excessive alcohol intake (>20 g/day in men and >10 g/day in women) and negative markers of hepatitis B and C. Because steatosis can reduce or disappear during advanced fibrosis or cirrhosis development (22–25), and our study includes NAFLD patients retrospectively from multicenter using ultrasonography to establish diagnosis, part of NAFLD patients would progress to inflammation and fibrosis stage with steatosis degree decreased and they might be misclassified as cryptogenic cirrhosis due to the insufficient sensitivity of ultrasound, therefore cryptogenic cirrhosis would be ascribed to mild NAFLD in the analysis to avoid underestimation in this study. And we repeated our primary analyses after re-matching NAFLD patients by excluding cryptogenic cirrhosis, to address potential confounding. We excluded patients with secondary causes of fatty liver (e.g., long-term consumption of the steroids amiodarone, tamoxifen or methotrexate). Each death record was based on information extracted from the electronic database systems in the participating centers. The death certificates were derived from these death records from hospitals and confirmed by two physicians (X. G and X. L) that were blinded to the aim of this study independently according to the 10th revision of the International Statistical Classification of Diseases (ICD-10) codes, as recommended by the World Health Organization (WHO) (Supplementary Table 1). The contributing causes of death in this study were determined to be NAFLD, other liver diseases (i.e., viral hepatitis, autoimmune liver diseases, congenital liver diseases, hemochromatosis, iron overload, Wilson's disease, cholangitis, Budd-Chiari syndrome, and drug injury) and no liver disease (Levels of liver functions were within the normal range at the last admission before death, and the patients were without established diagnose of causes of liver disease according to the medical records from the hospitals). Causes of death were classified into liver-related, extrahepatic neoplasms, cardio- and cerebrovascular, infectious, respiratory, digestive, renal, endocrine diseases, and others, while liver-related deaths were defined as hepatocellular carcinoma (HCC) and cirrhotic complications, in accordance with the ICD-10. Metabolic syndrome was based on International Diabetes Federation (2006) criteria.

### Clinical Evaluation

Baseline information collected during hospitalization but before treatment, including sex, age, race, and date of death, was extracted. Patient history data included demographics; past disorders; medication history; nicotine and alcohol consumption; and anthropometric measurements including body weight, body height, and blood pressure. Body mass index (BMI) was defined as the body weight in kilograms divided by the square of the body height in meters.

Serological examination data, including alanine transaminase (ALT), aspartate transaminase (AST), gamma-glutamyl transpeptidase (GGT), alkaline phosphatase (ALP), total cholesterol, triglycerides, high-density lipoprotein (HDL) cholesterol, low-density lipoprotein (LDL) cholesterol, fasting blood glucose (FBG), and uric acid, were assayed as mentioned previously. Metabolic syndrome is based on presence of at least 3 of 5 factors, which include triglycerides (TG) 150 mg/dL or greater, HDL cholesterol <40 mg/dL in men and <50 mg/dL in women, hypertension defined as systolic blood pressure 130 mmHg or greater or diastolic blood pressure 85 mmHg or greater, hyperglycemia defined as fasting glucose 100 g/dL or greater, and body mass index 25 kg/m^2^ or greater (26). Fibrosis index based on the 4 factor (FIB-4) was calculated as FIB-4 = age(year)^*^AST(U/L)/PLT(10^9^/L)^*^ALT(U/L)^0.5^ (27).

### Radiologic Assessment and Steatosis Grading

Fatty liver was evaluated with abdominal ultrasonography measurements within 1 year before death by experienced radiologists based on the following criteria: the presence of liver and kidney echo discrepancies, with or without the presence of posterior attenuation of the ultrasound beam, vessel blurring, and difficult visualization of the gallbladder wall and the diaphragm. Hepatic steatosis was further graded into 3 categories as follows: (1) mild steatosis, defined by the manifestation of diffusely increased echogenicity or hepatorenal contrast; (2) moderate steatosis, defined by the visualization of bright echoes and increased hepatorenal contrast concurrently; and (3) severe steatosis, defined by the observation of ultrasound beam attenuation based on the establishment of moderate steatosis.

### Statistical Analysis

Normally distributed data are depicted as medians (standard deviations). The Kruskal-Wallis rank sum test was applied to abnormally distributed continuous variables between groups. Chi-square tests were used for comparisons of categorical data between groups, with Bonferroni *post-hoc* tests for multiple comparisons among subgroups. Logistic regression models with stepwise selection were used to estimate odds ratios (OR) for the different degrees of NAFLD in relation to the causes of death. A two-tailed *P* < 0.05 was considered indicative of statistical significance. All data were analyzed using SPSS software (version 20.0, SPSS Inc., Chicago, IL, USA).

## Results

Between 2009 and 2018, there were 14,284 registered deaths during hospitalization. Ultrasound graphic assessment was performed in 10,071 cases (61.2% males, aged 64.8 ± 17.8 years): 2,015 NAFLD (20.0%, including 25 cases with cryptogenic cirrhosis), 1,140 other livers diseases (11.3%), and 6,916 no liver disease (68.7%), respectively. Lowest percentage of male appeared in the NAFLD groups (54.5%, *P* < 0.001), with the highest rate in the other liver disease group (71.8%), followed by no liver disease group (61.4%). Notedly, significant difference also existed in the mean age among the three groups was found (summarized in Table 1), with NAFLD and no liver diseases patients presenting a higher mean age to those with other liver diseases (64.4 ± 18.9 vs. 66.1 ± 19.2 vs. 57.4 ± 16.3 years, respectively). The NAFLD group presented higher BMI, systolic blood pressure (SBP), gamma-glutamyl transferase (GGT), total cholesterol, triglycerides, HDL cholesterol, LDL cholesterol, apolipoprotein-B, lipoprotein-A, fasting blood glucose, and uric acid but lower apolipoprotein-A1 levels than the no liver disease group (Table 1).

**Table 1:** Baseline characteristics of the deaths with NAFLD and non-NAFLD.

**Characteristics**	**NAFLD (*n* = 2,015)**	**Other liver diseases (*n* = 1,140)**	**No liver diseases (*n* = 6,916)**	***P***	***Post-hoc***		
					**NA vs. OL**	**NA vs. NL**	**OL vs. NL**
Male, *n* (%)	1,099 (54.5)	819 (71.8)	4,248 (61.4)	<0.001	<0.001	0.015	0.003
Age, year	64.4 ± 18.9	57.4 ± 16.3	66.1 ± 19.2	<0.001	<0.001	0.25	<0.001
BMI, kg/m^2^	24.3 ± 5.5	21.4 ± 3.2	21.6 ± 3.6	<0.001	<0.001	<0.001	0.670
SBP, mmHg	133.9 ± 23.2	125.4 ± 24.7	128.7 ± 25.4	0.002	<0.001	0.016	0.063
DBP, mmHg	79.7 ± 14.3	75.1 ± 15.5	75.1 ± 13.5	0.080	–	–	–
ALT, U/L^a^	31 (16–50)	51 (25–80)	31 (17–52)	<0.001	<0.001	0.32	<0.001
AST,U/L^a^	44 (24–71)	108 (69–168)	49 (25–83)	<0.001	<0.001	0.26	<0.001
GGT, U/L^a^	64 (31–104)	63 (32–103)	53 (26–96)	<0.001	0.81	0.001	0.005
ALP, U/L^a^	92 (66–141)	114 (76–171)	94 (66–121)	<0.001	0.001	0.59	<0.001
Total cholesterol, mmol/L	5.28 ± 1.29	2.70 ± 1.24	3.53 ± 1.17	<0.001	<0.001	<0.001	<0.001
Triglycerides, mmol/L	2.86 ± 1.44	0.99 ± 0.46	1.11 ± 0.48	<0.001	<0.001	<0.001	0.950
HDL-cholesterol, mmol/L	0.95 ± 0.32	0.63 ± 0.25	0.88 ± 0.23	<0.001	<0.001	0.006	<0.001
LDL-cholesterol, mmol/L	3.28 ± 1.55	1.69 ± 0.88	2.21 ± 0.86	<0.001	<0.001	<0.001	<0.001
Apolipoprotein-A1, g/L	0.64 ± 0.35	1.00 ± 0.38	0.90 ± 0.30	<0.001	<0.001	<0.001	<0.001
Apolipoprotein-B, g/L	0.97 ± 0.36	0.59 ± 0.25	0.71 ± 0.22	<0.001	<0.001	<0.001	<0.001
Lipoprotein-A, mg/L	353.3 ± 42.5	161.2 ± 23.7	289.3 ± 32.3	<0.001	<0.001	0.007	<0.001
FBG, mmol/L	7.9 ± 2.3	6.4 ± 1.5	7.1 ± 2.0	<0.001	<0.001	<0.001	0.014
Uric acid, umol/L	368.7 ± 40.6	302.2 ± 30.1	334.4 ± 32.4	<0.001	<0.001	0.027	0.081

*BMI, body mass index; SBP, systolic blood pressure; DBP, diastolic blood pressure; ALT, alanine aminotransferase; AST, aspartate aminotransferase; GGT, gammaglutamyl transpeptidase; ALP, alkaline phosphatase; HDL-cholesterol, high-density lipoprotein-cholesterol; LDL-cholesterol, low-density lipoprotein-cholesterol; FBG, fasting blood glucose*.

a *Continuous variables are expressed as median with 25th−75th interquartile range for non-Gaussian distribution*.

### Mortality Trends of NAFLD

**Figure 1:**
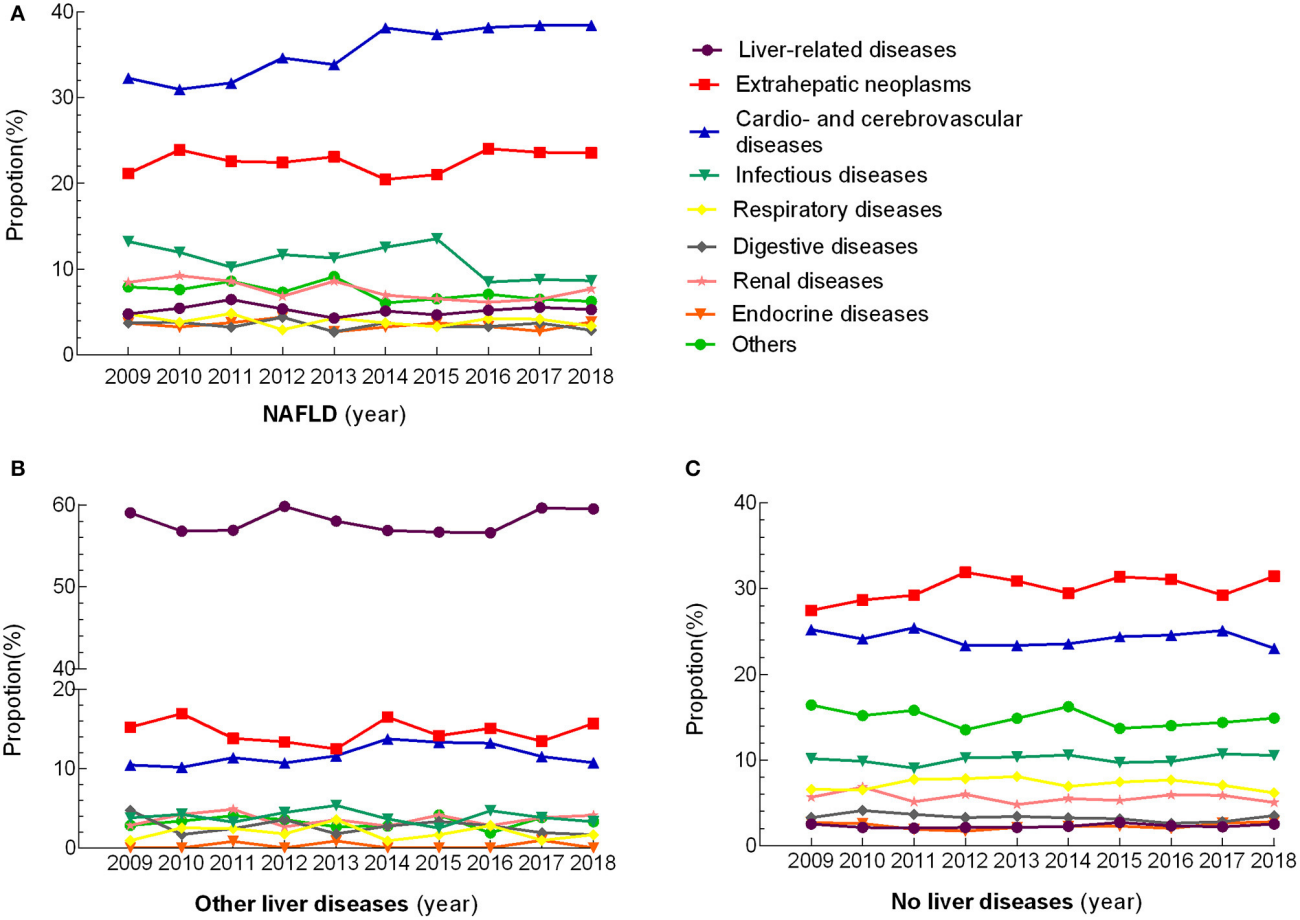
Changing trends in the proportion of cause of death over 10 consecutive years. Every marker illustrated the proportion of death due to a different cause that was indicated as different colored lines.

The distribution of death causes remained steady in the three groups over the decade (Figure 1). Within the NAFLD subgroups, the leading causes of mortality were categorized as CVDs (35.6%), followed by extrahepatic tumors (22.6%), infectious diseases (11.0%), kidney disease (7.5%), other diseases (7.2%), liver-related diseases (5.2%), respiratory diseases (3.9%), digestive diseases (3.5%), and endocrine diseases (3.5%). A higher proportion of ischemic heart diseases was noted in NAFLD patients than in patients with other liver disease and without liver disease (21.6 vs. 4.0 vs. 14.7%, *P* < 0.001), and similar trend presented in cerebrovascular disease (7.5 vs. 4.1 vs. 5.2%) and chronic heart disease (4.3 vs. 1.2 vs. 2.9%). Patients in the NAFLD group died from liver-related diseases significantly more frequently than those without liver diseases (5.2 vs. 2.3%, *P* < 0.001), but much less than those with other liver diseases (58.3%, *P* < 0.001). For extrahepatic tumors, there was a stepwise increase among the other liver disease group, NAFLD group and no liver disease group (14.6 vs. 22.6 vs. 30.1%, *P* < 0.001); however, for specific cancer types, colorectal cancer was overrepresented in with NAFLD than those with no liver diseases (6.2 vs. 5.0%, *P* = 0.034), while opposite trend was shown in lung (3.1 vs. 5.2%, *P* < 0.001), and stomach cancer (2.5 vs. 4.1%, *P* = 0.002), and no difference was found for hematologic cancer between these two groups (4.8 vs. 6.1%, *P* = 0.06). Among the deaths attributed to infectious diseases, pneumonia and sepsis were identified as the most prominent in the NAFLD patients; they were more common in NAFLD patients than in those with other liver diseases and similar in those without liver diseases (6.7 vs. 2.6 vs. 6.3%) (Table 2). Analyzing the data according to sex showed that deaths attributed to liver-related diseases occurred more frequently in males (*P* < 0.001) and deaths attributed to endocrine diseases occurred more frequently in females (*P* < 0.001) in the NAFLD group, while the other causes of death showed no significant sex differences; this trend was similar in the other liver diseases group (*P* < 0.001 for liver-related diseases and *P* = 0.006 for endocrine diseases). Sex difference was found for those without liver diseases in any of the subgroups except for the infectious disease (Supplementary Table 2).

**Table 2:** Cause of deaths in patients with NAFLD vs. non-NAFLD.

**Diseases, *n* (%)**	**NAFLD (*n* = 2,015)**	**Other liver diseases (*n* = 1,140)**	**No liver diseases (*n* = 6,916)**	***P***	***Post-hoc***		
					**NA vs. OL**	**NA vs. NL**	**OL vs. NL**
**Liver-related diseases**	105 (5.2)	665 (58.3)	159 (2.3)	<0.001	<0.001	<0.001	<0.001
Hepatocellular carcinoma	51 (2.5)	327 (28.7)	104 (1.5)	<0.001	<0.001	0.004	<0.001
Decompensated cirrhosis	22 (1.1)	216 (18.9)	21 (0.3)	<0.001	<0.001	<0.001	<0.001
Hepatic failure	32 (1.6)	122 (10.7)	34 (0.5)	<0.001	<0.001	<0.001	<0.001
**Extrahepatic neoplasms**	455 (22.6)	166 (14.6)	2,082 (30.1)	<0.001	<0.001	<0.001	<0.001
Colorectal cancer	125 (6.2)	45 (3.9)	346 (5.0)	0.024	0.013	0.034	0.190
Lung cancer	62 (3.1)	30 (2.6)	360 (5.2)	<0.001	0.570	<0.001	<0.001
Stomach cancer	50 (2.5)	25 (2.2)	284 (4.1)	<0.001	0.710	0.002	0.006
Hematologic malignancy	97 (4.8)	41 (3.6)	422 (6.1)	0.001	0.310	0.060	0.004
Other malignancies	121 (6.0)	25 (2.2)	670 (9.7)	<0.001	<0.001	<0.001	<0.001
**Cardio- and cerebrovascular diseases**	717 (35.6)	132 (11.6)	1,674 (24.2)	<0.001	<0.001	<0.001	<0.001
Ischemic heart disease	436 (21.6)	46 (4.0)	1,017 (14.7)	<0.001	<0.001	<0.001	<0.001
Cerebrovascular disease	151 (7.5)	47 (4.1)	360 (5.2)	<0.001	<0.001	<0.001	0.374
Chronic heart disease	86 (4.3)	14 (1.2)	200 (2.9)	<0.001	<0.001	0.004	0.004
Others	44 (2.2)	25 (2.2)	97 (1.4)	0.013	0.880	0.026	0.058
**Infectious diseases**	222 (11.0)	44 (3.9)	699 (10.1)	<0.001	<0.001	0.470	<0.001
Pneumonia or sepsis	136 (6.7)	30 (2.6)	436 (6.3)	<0.001	<0.001	0.950	<0.001
Aspergillosis	58 (2.9)	8 (0.7)	187 (2.7)	<0.001	<0.001	0.670	<0.001
Tuberculosis	12 (0.6)	3 (0.3)	41 (0.6)	0.402	–	–	–
Others	16 (0.8)	3 (0.3)	35 (0.5)	0.132	–	–	–
**Respiratory diseases**	79 (3.9)	22 (2.0)	498 (7.2)	<0.001	0.008	<0.001	<0.00
Chronic obstructive pulmonary diseases	50 (2.5)	11 (1.0)	325 (4.7)	<0.001	0.008	<0.001	<0.001
Pulmonary arterial hypertension	16 (0.8)	5 (0.5)	104 (1.5)	0.002	0.530	0.030	0.010
Lung fibrosis	5 (0.2)	3 (0.3)	27 (0.4)	0.570	–	–	–
Others	8 (0.4)	3 (0.3)	42 (0.6)	0.240	–	–	–
**Digestive diseases**	70 (3.5)	30 (2.7)	228 (3.3)	0.520	–	–	–
Pancreatitis	38 (1.9)	15 (1.4)	124 (1.8)	0.580	–	–	–
Upper gastrointestinal hemorrhage	24 (1.2)	11 (1.0)	83 (1.2)	0.840	–	–	–
Others	8 (0.4)	4 (0.4)	21 (0.3)	0.800	–	–	–
**Renal diseases**	151 (7.5)	41 (3.7)	387 (5.6)	<0.001	<0.001	0.004	0.020
Chronic kidney diseases	78 (3.9)	23 (2.1)	214 (3.1)	0.020	0.014	0.170	0.130
Acute kidney injury	59 (2.9)	15 (1.4)	138 (2.0)	0.007	0.012	0.024	0.310
Others	14 (0.7)	3 (0.3)	35 (0.5)	0.280	–	–	–
**Endocrine diseases**	70 (3.5)	3 (0.3)	159 (2.3)	<0.001	<0.001	0.006	<0.001
Diabetes mellitus	46 (2.3)	2 (0.2)	97 (1.4)	<0.001	<0.001	0.012	0.002
Thyrotoxicosis	15 (0.7)	1 (0.1)	41 (0.6)	0.060	–	–	–
Others	9 (0.4)	0 (0)	21 (0.3)	0.091	–	–	–
**Others**	146 (7.2)	37 (3.4)	1,030 (14.9)	<0.001	<0.001	<0.001	<0.001

*NA, NAFLD; OL, Other liver diseases; NL, No liver diseases*.

*Chi-squared test for the comparison of cause of deaths distribution between NAFLD and non-NAFLD*.

### Obesity and Causes of Death in NAFLD

To examine the effect of obesity on cause of death, we stratified the patients into obese and non-obese group, defined based on BMI ≥25 and <25 kg/m^2^, respectively. Deaths attributed to cardio- and cerebrovascular diseases occurred more frequently in the obese with NAFLD (35.6 vs. 30.3%, *P* < 0.001), while opposite trend was shown in NAFLD group dying from infectious diseases (11.1 vs. 13.4%, *P* < 0.001) and renal diseases (6.1 vs. 8.7%, *P* = 0.027). The other causes of death showed no significant differences in term of BMI (Supplementary Table 3).

### Associations Among Steatosis Grade, Advanced Fibrosis and Causes of Mortality

In the subjects with NAFLD, the degree of hepatic steatosis was further assessed by abdominal ultrasound as mild (565 cases, 28.0%), moderate (1,102 cases, 54.7%), or severe (348 cases, 17.3%). After multiple adjustments for sex, body mass index, fasting blood glucose, low-density lipoprotein cholesterol, uric acid, metabolic syndrome, and FIB-4 index, the binary logistic regression analysis indicated that the steatosis degree increase was associated with an increased risk of death from liver-related diseases (OR = 1.37, 95% CI: 1.20–1.59, *P* < 0.001), cardio- and cerebrovascular diseases (OR = 1.23, 95% CI: 1.19–1.31, *P* < 0.001), infectious diseases(OR = 1.14, 95% CI: 1.04–1.26, *P* = 0.035), and renal diseases (OR = 1.21, 95% CI: 1.02–1.47, *P* = 0.043), while the other causes of death showed no significant association with NAFLD severity (Table 3). In NAFLD group, subjects with advanced fibrosis predicted by FIB-4 (cut-off value = 1.3) significantly more frequently died from liver-related diseases (*P* < 0.001), extrahepatic neoplasms (*P* = 0.043), cardio- and cerebrovascular diseases (*P* = 0.038), and infectious diseases (*P* = 0.010) (Supplementary Table 4).

**Table 3:** Association between steatosis degrees and distribution of causes of death.

**Diseases, *n* (%)**	**Non-NAFLD (*n* = 6,916)**	**Mild (*n* = 565)**	**Moderate (*n* = 1,102)**	**Severe (*n* = 348)**	**Univariate**		**Multivariate**	
					**OR (95% CI)**	***P***	**OR (95% CI)**	***P***
Liver-related diseases	159 (2.3)	24 (4.2)	61 (5.5)	20 (5.7)	1.35 (1.17–1.57)	<0.001	1.37 (1.20–1.59)	<0.001
Extrahepatic neoplasms	2,082 (30.1)	182 (32.2)	228 (20.7)	45 (12.9)	0.92 (0.82–0.99)	0.001	0.96 (0.83–1.02)	0.160
Cardio- and cerebrovascular diseases	1,674 (24.2)	209 (37.0)	394 (35.8)	114 (32.8)	1.22 (1.16–1.30)	<0.001	1.23 (1.19–1.31)	<0.001
Infectious diseases	699 (10.1)	56 (9.9)	104 (9.4)	62 (17.8)	1.16 (1.02–1.36)	0.025	1.14 (1.04–1.26)	0.035
Respiratory diseases	498 (7.2)	28 (4.9)	40 (3.6)	11 (3.2)	0.86 (0.79–0.96)	0.020	0.89 (0.78–0.98)	0.250
Digestive diseases	228 (3.3)	18 (3.2)	35 (3.2)	17 (4.9)	1.03 (0.81–1.36)	0.780	1.05 (0.79–1.38)	0.680
Renal diseases	387 (5.6)	18 (3.2)	110 (10.0)	23 (6.6)	1.19 (1.01–1.45)	0.045	1.21 (1.02–1.47)	0.043
Endocrine diseases	159 (2.3)	11 (1.9)	43 (3.9)	16 (4.6)	1.18 (0.92–1.57)	0.540	1.15 (0.89–1.54)	0.440
Others	1,030 (14.9)	19 (3.4)	87 (7.9)	40 (11.5)	0.89 (0.79–1.01)	0.051	0.88 (0.77–0.99)	0.042

FIB-4, fibrosis index based on the 4 factor; FIB-4 = age (year)^*^AST (U/L)/PLT (10^9^/L) ^*^ALT (U/L)^0^.5.

*Multivariate logistics regression analysis adjusted for sex, body mass index, fasting blood glucose, low-density lipoprotein cholesterol, uric acid metabolic syndrome and FIB-4. Non-NAFLD group was used as reference for this analysis*.

### Characteristics of Different Causes of Deaths With NAFLD

The anthropometrical and metabolic characteristics of different causes of death in the NAFLD group are presented in Table 4. Significant differences in age, sex, BMI, and SBP or diastolic blood pressure (DBP) were found among the different causes. Regarding hepatic markers and metabolic characteristics among the different causes, ALT, AST, ALP, total cholesterol, triglycerides, HDL cholesterol, LDL cholesterol, apolipoprotein-A1, and lipoprotein-A showed significant differences, while GGT, FBG, uric acid and apolipoprotein-B did not show significant differences.

**Table 4:** Anthropometrical and metabolic characteristics of different causes of deaths with NAFLD.

**Characteristics**	**Liver-related diseases (*n* = 105)**	**Extrahepatic neoplasms (*n* = 455)**	**Cardio-and cerebrovascular diseases (*n* = 717)**	**Infectious diseases (*n* = 222)**	**Respiratory diseases (*n* = 79)**	**Digestive diseases (*n* = 70)**	**Renal diseases (*n* = 151)**	**Endocrine diseases (*n* = 70)**	**Others (*n* = 146)**	***P***
Male, *n* (%)	78 (74.3) bcdefgh	242 (53.2) ahi	401 (55.9) ahi	136 (61.3) aghi	47 (59.5) ahi	35 (50.0) adhi	77 (51.0) adhi	23 (32.9) abcdefg	58 (39.7) abcdefg	<0.001
Age, year	54.0 ± 17.8 bcdefgh	65.5 ± 16.4 aghi	70.0 ± 15.8 agi	66.4 ± 19 agi	64.9 ± 18.6 aghi	64.5 ± 19.6 aghi	59.2 ± 21.0 abcdefh	71.2 ± 14.0 abefgi	56.8 ± 20.3 bcdefh	<0.001
BMI, kg/m^2^	27.1 ± 3.8 bg	22.8 ± 4.2 afh	24.4 ± 4.8 gh	24.6 ± 6.3 gh	25.3 ± 7.8 gh	25.8 ± 5.0 bgh	21.2 ± 3.3 acdefhi	30.4 ± 8.2 bcdefgi	24.4 ± 4.7 gh	0.038
SBP, mmHg	130.5 ± 16.0 cd	129.4 ± 10.5 eghi	149.3 ± 21.8 abdefhi	129.6 ± 10.4 bcfgh	136.3 ± 19.0 abcfgh	130.6 ± 11.9 cdegh	148.8 ± 23.6 abdefhi	159.0 ± 18.1 abcdefgi	133.0 ± 10.8 cgh	<0.001
DBP, mmHg	80.6 ± 8.2 gh	75.9 ± 7.2 cefgh	85.8 ± 9.8 bdi	75.7 ± 8.9 cfgh	79.3 ± 7.3 bgh	83.0 ± 9.6 bdghi	89.0 ± 9.2 abdefi	88.7 ± 9.9 abdefi	77.8 ± 8.1 cfgh	<0.001
ALT, U/L^†^	55 (33–65) bcdefghi	29 (16–43) adg	29 (16–46) adg	41 (17–65) abcefghi	27 (16–38) adg	23 (14–40) adghi	20 (9–28)a bcdefhi	32 (11–50)adfg	33 (15–50) adfg	<0.001
AST, U/L^†^	112 (67–160) bcdefghi	49 (24–73)acdefg	38 (23–53)abdhi	60 (28–88) abcefghi	37 (24–50) abdhi	33 (22–44)abdhi	30 (19–45) abdhi	48 (24–80)acdefg	47 (25–72)acdefg	<0.001
GGT, U/L^†^	101 (35–158)	73 (31–110)	56 (30–78)	74 (35–105)	53 (32–75)	51 (26–75)	43 (30–55)	53 (22–78)	69 (30–89)	0.330
ALP, U/L^†^	187 (113–255) bcdefghi	113 (68–154) acdeghi	82 (63–101) abdefi	130 (83–167) abcefghi	89 (68–110) abcdfg	111 (67–150) acdeghi	74 (53–100) abdefhi	84 (63–105) abdfg	92 (65–118) abcdfg	<0.001
Total cholesterol, mmol/L	5.7 ± 1.4defi	5.7 ± 1.4defi	5.5 ± 1.0 dei	4.3 ± 1.0abcefghi	5.0 ± 0.9 abcdfgh	3.7 ± 1.2 abdeghi	5.5 ± 1.1 defi	5.5 ± 1.5 defi	4.8 ± 1.3 abcdfgh	0.001
Triglycerides, mmol/L	2.5 ± 1.5 bdefgi	2.7 ± 1.0 acdhi	2.5 ± 1.2 bdefgi	3.8 ± 1.2 abcefh	2.8 ± 1.0 acdhi	3.0 ± 1.1 acdhi	3.4 ± 1.5 ach	2.4 ± 1.4 bdefgi	3.8 ± 2.4 abcefh	0.006
HDL-cholesterol, mmol/L	0.71 ± 0.17bceg	1.04 ± 0.25 adfi	1.08 ± 0.22 adfi	0.74 ± 0.14 bcef	1.22 ± 0.22adfi	0.54 ± 0.15 bcdeghi	0.99 ± 0.21 afi	1.06 ± 0.22 dfi	0.74 ± 0.12 bcefgh	<0.001
LDL-cholesterol, mmol/L	3.62 ± 1.19dfi	3.55 ± 1.29 dfi	3.45 ± 1.09 dfi	2.50 ± 1.1 abcegh	3.50 ± 1.17 dfi	2.24 ± 0.52abcegh	3.26 ± 1.61 df	3.55 ± 1.14 dfi	2.84 ± 0.64 abceh	<0.001
Apolipoprotein-A1, g/L	0.65 ± 0.36cegh	0.97 ± 0.51 cef	1.16 ± 0.44 abdfi	0.79 ± 0.45 cefgh	1.24 ± 0.48 abdfi	0.6 ± 0.24bcdeghi	1.08 ± 0.44 adf	1.12 ± 0.35 adf	0.81 ± 0.43 cef	<0.001
Apolipoprotein-B, g/L	0.91 ± 0.39	1.03 ± 0.35	0.99 ± 0.34	0.87 ± 0.28	0.97 ± 0.24	0.67 ± 0.40	0.97 ± 0.24	1.06 ± 0.26	1.05 ± 0.25	0.150
Lipoprotein-A, mg/L	234.8 ± 36.5 bcdeghi	256.9 ± 39.9 acefghi	438.3 ± 60.0 abdefi	262.6 ± 63.7 acefghi	329.0 ± 53.5 abcdfgh	207.3 ± 64.1 bcdeghi	446.3 ± 33.2 abdefi	430.1 ± 46.2 abdefi	329.3 ± 63.1 abcdfgh	0.047
FBG, mmol/L	7.5 ± 1.6	8.3 ± 1.7	8.8 ± 1.8	8.6 ± 2.2	7.7 ± 1.0	8.0 ± 1.8	7.3 ± 1.5	9.1 ± 2.5	7.7 ± 2.1	0.310
Uric acid, umol/L	380.0 ± 39.4	390.3 ± 37.0	414.6 ± 38.9	383.9.0 ± 33.5	442.5 ± 38.8	385.2 ± 38.3	415.5 ± 50.6	339.0 ± 38.5	358.9 ± 38.3	0.270

*BMI, body mass index; SBP, systolic blood pressure; DBP, diastolic blood pressure; ALT, alanine aminotransferase; AST, aspartate aminotransferase; GGT, gamma glutamyl transpeptidase; ALP, alkaline phosphatase; HDL-cholesterol, high-density lipoprotein-cholesterol; LDL-cholesterol, low-density lipoprotein-cholesterol; FBG, fasting blood glucose*.

† *Continuous variables are expressed as median with IQR for non-Gaussian distribution. P values were for the ANOVA analysis across the groups, different*.

*a, b, c, d, e, f, g, h, i- refer to statistic significant after post-hoc multiple comparisons with Bonferroni adjustments when compared with Liver-related diseases group (a), Extrahepatic neoplasms group (b), Cardio-and cerebrovascular diseases group (c), Infectious diseases group (d), Respiratory diseases group (e), Digestive diseases group (f), Renal diseases group (g), Endocrine diseases group (h), and Others (i)*.

### Relation Between NAFLD and Multiple Organ Dysfunction Syndrome (MODS)

The occurrence of multiple organ dysfunction syndrome (MODS) among the three groups during death was calculated. A total of 877 cases of heart failure (43.5%), followed by respiratory failure (43.9%), renal failure (32.5%), hepatic failure (13.2%), and disseminated intravascular coagulation (12.6%) occurred in the NAFLD group, with all the organ dysfunctions being more prevalent in the NAFLD group than the other two groups except for the hepatic failure. NAFLD patients had significantly higher prevalence rates of two organ dysfunctions (15.5 vs. 9.5%, *P* < 0.001) than the no liver disease group, but these rates were not significantly different from those in the other liver disease group (*P* = 0.09). Finally, the presence of MODS was highest (29.6 vs. 25.8% in other liver disease vs. 21.6% in no liver disease, *P* < 0.001) than that in the other groups (Table 5).

**Table 5:** The occurrence of multiple organ dysfunction syndrome (MODS) in patients with NAFLD vs. non-NAFLD.

**Organ dysfunction, *n* (%)**	**NAFLD (*n* = 2,015)**	**Other liver diseases (*n* = 1,140)**	**No liver diseases (*n* = 6,916)**	***P***	***Post-hoc***		
					**NA vs. OL**	**NA vs. NL**	**OL vs. NL**
Heart failure	877 (43.5)	412 (36.1)	2,185 (31.6)	<0.001	<0.001	<0.001	0.006
Respiratory failure	885 (43.9)	405 (35.5)	2,248 (32.5)	<0.001	<0.001	<0.001	0.102
Renal failure	655 (32.5)	259 (22.7)	1,480 (21.4)	<0.001	<0.001	<0.001	1.000
Hepatic failure	266 (13.2)	312 (27.4)	539 (7.8)	<0.001	<0.001	<0.001	<0.001
Disseminated intravascular coagulation	254 (12.6)	119 (10.4)	387 (5.6)	<0.001	0.146	<0.001	<0.001
Two organ dysfunctions	312 (15.5)	130 (11.4)	657 (9.5)	<0.001	0.004	<0.001	0.090
MODS	596 (29.6)	294 (25.8)	1,494 (21.6)	<0.001	0.048	<0.001	0.004

*NA, NAFLD; OL, Other liver diseases; NL, No liver diseases; MODS, multiple organ dysfunction syndrome*.

*Chi-squared test for the occurrence of organ dysfunction, which included single, two and multiple organ dysfunction between NAFLD and non-NAFLD*.

### Sensitivity Anlyses

Data using another NAFLD subset which censored cryptogenic cirrhosis was exhibited in Supplementary Tables 5–12). The results were similar to that using cryptogenic cirrhosis as definition of mild NAFLD, and we observed a similar, dose-dependent association between steatosis degree and specific death causes proportions, as well as increased MODS.

## Discussion

Liver disease is the 12th leading cause of death worldwide, and there is a substantial increase related to NAFLD in liver-related mortality (16). Using 10 years of regional representative mortality data with over 10,000 samples in southern China, we presented a comprehensive analysis of NAFLD-specific mortality and compared it with mortality attributable to other liver diseases and no liver diseases. Our analysis revealed that NAFLD was associated with high rates of ischemic heart diseases and liver-related diseases. Death from pneumonia or sepsis was more frequent in NAFLD patients than in other liver disease patients. Moreover, the steatosis degree was also independently associated with liver-related diseases and cardio- and cerebrovascular diseases. In addition, the presence of NAFLD was a significant risk factor for multisystem failure in patients who died during hospitalization.

One of the important findings of our study was that NAFLD was associated with the burden of CVD, with a more than 40% increase in the mortality ratio for CVD among all deaths, and the severity of steatosis independently correlated with these relationships in the Chinese population. It has been recognized that CVDs are the primary cause of mortality in patients with NAFLD; however, results regarding the relationship between NAFLD and the increased risk of clinical cardiovascular events are conflicting. A recent meta-analysis of 25,837 patients from a six-cohort study demonstrated that the risk of clinical cardiovascular events was significantly higher in patients with NAFLD than in those without NAFLD (RR: 1.77; 95% CI: 1.26–2.48, *P* < 0.001), with a similar shift in both cardiovascular mortality and stroke after subgroup analysis (18). In a more recent longitudinal study with a 10-year follow-up in southern Asia, a lack of an association between NAFLD and cardiovascular mortality was reported (19). Another prospective cohort study of 612 patients with clinical indications for coronary angiography in Hong Kong showed that fatty liver could not predict death due to CVD (28). The heterogeneous conclusions among studies can be attributed to several reasons. First, the endpoints in the other studies focused on the incidence instead of the mortality attributable to CVD in NAFLD participants (29–31). Using CVD-related mortality as an outcome may reveal a final prognosis of NAFLD in the real world. Moreover, the increased risk of CVD caused by NAFLD is modified by disease extent, such as fibrosis staging or pathologic scores. Our research included the death cases as the study subjects, and it could be assumed that these patients had a more pronounced progression stage than those in cohort studies. Furthermore, metabolic abnormalities are strong cardiovascular risk factors, and the high collection of metabolic risk factors among patients with NAFLD might also account for the increase in coronary artery disease (CAD)-related deaths. Thus, our results indicate that aggressive treatment of NAFLD and other cardiovascular risk factors might be warranted.

Extra liver cancer was the second most common cause of death in our cohort, and among patients with NAFLD, a 12% increased risk of extra liver cancer was observed. This was similar to observations in other studies across different geographical areas and ethnicities. A recent longitudinal cohort study from the United States with over 20 years of follow-up and more than 10,000 individuals demonstrated that NAFLD was associated with a nearly 2-fold increased risk of developing cancer (13), with the highest risks for uterine (IRR = 2.3), stomach (IRR = 2.3), pancreatic (IRR = 2.1), and colon cancers (IRR = 1.8). The varied correlation between NAFLD and these types of cancer in our study was mainly due to the limited sample size, population characteristics and cross-sectional study design, and especially a bias caused by death unrelated to cancer. Collectively, these results suggest that the relationship between NAFLD and the clinical burden of cancer warrants further research.

The third most common cause of death among NAFLD patients was liver-related deaths, according to previous reports (20). As opposed to the Caucasian population, liver-specific death ranked as the sixth cause of mortality in our studied population. The considerable discordance in the ratio of liver-related outcomes between populations in China and developed countries may result from variations in nutrition and lifestyle determinants that drive hepatic inflammation and fibrogenesis (32). Notably, Western diets contain much higher proportions of fat, fructose, and red meat than the traditional Chinese dietary pattern (characterized by refined grains, vegetables, pork, poultry, and fruits) (33). Western diets promote excess lipid genesis in hepatocytes and therefore induce lipoapoptosis and disease escalation. However, with the changes from a traditional agricultural society to an industrialized urbanized society in the past decades, modern Chinese dietary patterns tend to be similar to westernized diets in part. Therefore, the liver-related mortality rate associated with NAFLD in China might lag behind a shift in socioeconomic status.

When analyzing the characteristics of NAFLD-related deaths, we first observed that NAFLD patients suffered from a higher rate of multiple organ failure than those without liver disease and the end of life. It has been increasingly acknowledged that NAFLD may be accompanied by obesity, insulin resistance, diabetes and a chronic system inflammatory state, which has been linked to multiple complications, including community-acquired pneumonia, chronic obstructive pulmonary disease and kidney injuries (4, 5, 7). Inflammation plays a critical role in initiating and auto amplifying organ failure (34). Furthermore, a steatosis liver, especially in the non-alcoholic steatohepatitis (NASH) stage, releases several proinflammatory cytokines, such as interleukin-1, interleukin-6 and tumor necrosis factors, involved in the activation of deleterious inflammatory pathways. Thus, our data provide important evidence that NAFLD might be an additional predictor of the development of multiorgan failure, and intensive prevention should be implemented.

The limitations of our study should be noted. Our retrospective design and hospital-based data may introduce unavoidable bias. It is possible that in clinical practice many hospitalized patients were not routinely evaluated with any liver imaging during hospitalization. Thus, selection bias for the categorization of the study groups highly potential developed in this study. Second, the cross-sectional analysis could not identify the impact of the progression or regression of NAFLD on disease-specific death causes. Third, to evaluate the effect of steatosis on the distribution of cause of death, we used ultrasonographic features for the grading of hepatic steatosis, which were interpreted by several sonographers from the medical records. That might cause interpersonal variability for interpretation of liver findings. Another major drawback was that we used US to diagnose and stratify the severity of NAFLD, without histological confirmation. However, the histological confirmation of NAFLD in a large general population may be impractical.

## Conclusion

In conclusion, our large, regional survey showed that patients with NAFLD in China had a distinct mortality pattern. High mortality attributable to CVD and multiorgan failure occurred in NAFLD patients. Controlling NAFLD may provide benefits in lowering the burden of associated compilations.

## Data Availability Statement

The raw data supporting the conclusions of this article will be made available by the authors, without undue reservation.

## Ethics Statement

The studies involving human participants were reviewed and approved by Clinical Research Ethics Committee of the First Affiliated Hospital of Sun Yat-sen University, the First Affiliated Hospital of Guangzhou Medical University, and the Affiliated Dongguan People's Hospital of Southern Medical University. The patients/participants provided their written informed consent to participate in this study. Written informed consent was obtained from the individual(s) for the publication of any potentially identifiable images or data included in this article.

## Author Contributions

BZ and JY: conceive, design, and critical revision of the manuscript for important intellectual content. YL, XG, and XL: data collection, analysis, and manuscript drafting. ML, TW, CS, QM, and FL: data collection. All authors have read and approved the final manuscript.

## Conflict of Interest

The authors declare that the research was conducted in the absence of any commercial or financial relationships that could be construed as a potential conflict of interest.
