# Supplementary material for: Distinct Cause of Death Profiles of Hospitalized Non-alcoholic Fatty Liver Disease: A 10 Years' Cross-Sectional Multicenter Study in China
Source: Front Med (Lausanne). 2021 Jan 12;7:584396. doi: 10.3389/fmed.2020.584396 (PMC7835127; doi:10.3389/fmed.2020.584396)
Supplement: Supplementary file 1 [file Data_Sheet_1.docx]

Supplemental table 1. ICD-10 codes used to define disease groups

| Disease group | Individual disease name | Diagnosis/procedure code |
| --- | --- | --- |
| Liver-related diseases | 1. Hepatocellular carcinoma | C22.001 |
|  | 1. Decompensated cirrhosis | K74.151 |
|  | 1. Hepatic failure | K72.002, K72.051, K72.151 |
| Extrahepatic neoplasms | 1. Colorectal cancer | C19.X51 |
|  | 1. Lung cancer | C34.901 |
|  | 1. Stomach cancer | C16 |
|  | 1. Hematologic malignancy | C91, C92, C82 |
|  | 1. Renal cancer | C64.X01 |
|  | 1. Meningioma | D32 |
|  | 1. Neuroglioma | C71.003, C71.053, C71.055, C85.751 |
|  | 1. Bladder cancer | C67.901 |
|  | 1. Thyroid cancer | C73 |
|  | 1. Prostate cancer | C61 |
|  | 1. Uterine cancer | C55.X01, C53.902 |
|  | 1. Breast cancer | C50.902 |
|  | 1. Nasopharyngeal cancer | C11.901 |
| Cardio- and cerebro-  vascular diseases | 1. Ischemic heart disease | I20-I25 |
|  | 1. Cerebrovascular disease | I60-I69 |
|  | 1. Chronic heart disease | I50.911 |
| Infectious diseases | 1. Pneumonia or sepsis | J98.402 |
|  | 1. Aspergillosis | B44.901 |
|  | 1. Tuberculosis | A15.001 |
| Respiratory diseases | 1. Chronic obstructive pulmonary diseases | J44.901 |
|  | 1. Pulmonary arterial hypertension | I27.001 |
|  | 1. Lung fibrosis | J84.103 |
| Digestive diseases | 1. Pancreatitis | K85.X11 |
|  | 1. Upper gastrointestinal hemorrhage | K92.204 |
| Renal diseases | 1. [Chronic](D:/Program%20Files%20(x86)/Youdao/Dict/8.5.3.0/resultui/html/index.html#/javascript:;) [kidney](D:/Program%20Files%20(x86)/Youdao/Dict/8.5.3.0/resultui/html/index.html#/javascript:;) [diseases](D:/Program%20Files%20(x86)/Youdao/Dict/8.5.3.0/resultui/html/index.html#/javascript:;) | N18.807, N18.903 |
|  | 1. Acute kidney injury | N17.001 |
| Endocrine diseases | 1. [Diabetes](D:/Program%20Files%20(x86)/Youdao/Dict/8.5.3.0/resultui/html/index.html#/javascript:;) [mellitus](D:/Program%20Files%20(x86)/Youdao/Dict/8.5.3.0/resultui/html/index.html#/javascript:;) | E10 |
|  | 1. Thyrotoxicosis | E05.152 |

All deaths were coded on the basic of the 10^th^ revision of the International Classification of Diseases (ICD-10). Death causes that were not included in the list would be categorized as others.

Supplementary table 2. Cause of deaths in patients with NAFLD and non-NAFLD, stratified by sex

| Diseases, n(%) | NAFLD (n=2015) | | |  | Other liver diseases (n=1140) | | | No liver diseases (n=6916) | | | |
| --- | --- | --- | --- | --- | --- | --- | --- | --- | --- | --- | --- |
|  | Male  (n=1099) | Female  (n=916) | *P* |  | Male  (n=819) | Female  (n=321) | *P* |  | Male  (n=4248) | Female  (n=2668) | *P* |
| **Liver-related diseases**  diseases | 78(7.1) | 27(2.9) | <0.001 |  | 503(61.4) | 162(50.5) | <0.001 |  | 115(2.7) | 44(1.6) | 0.004 |
| Hepatocellular carcinoma | 39(3.5) | 12(1.3) | 0.001 |  | 256(31.3) | 71(22.1) | 0.002 |  | 75(1.8) | 29(1.1) | 0.024 |
| Decompensated cirrhosis | 15(1.4) | 7(0.8) | 0.196 |  | 163(19.9) | 53(16.5) | 0.189 |  | 15(0.4) | 6(0.2) | 0.345 |
| Hepatic failure | 24(2.2) | 8(0.9) | 0.019 |  | 84(10.3) | 38(11.8) | 0.758 |  | 25(0.6) | 9(0.3) | 0.146 |
| **Extrahepatic neoplasms** | 242(22.0) | 213(23.4) | 0.473 |  | 104(12.7) | 62(19.3) | 0.004 |  |  | 842(31.6) | 0.037 |
| Colorectal cancer | 72(6.6) | 53(5.8) | 0.478 |  | 28(3.4) | 17(5.3) | 0.143 |  | 207(4.9) | 139(5.2) | 0.531 |
| Lung cancer | 35(3.2) | 27(2.9) | 0.759 |  | 20(2.4) | 10(3.1) | 0.523 |  | 214(5.0) | 146(5.5) | 0.428 |
| Stomach cancer | 27(2.5) | 23(2.5) | 0.938 |  | 15(1.8) | 10(3.1) | 0.183 |  | 169(4.0) | 115(4.3) | 0.498 |
| Hematologic malignancy | 48(4.4) | 49(5.3) | 0.305 |  | 24(2.9) | 17(5.3) | 0.054 |  | 251(5.9) | 171(6.4) | 0.397 |
| Other malignancies | 60(5.5) | 61(6.7) | 0.259 |  | 17(2.1) | 8(2.5) | 0.666 |  | 399(9.4) | 271(10.2) | 0.295 |
| **Cardio- and cerebro- vascular diseases** | 401(36.5) | 316(34.5) | 0.331 |  | 94(11.5) | 38(11.8) | 0.864 |  | 1084(25.5) | 590(22.0) | 0.001 |
| Ischemic heart disease | 250(22.7) | 186(20.3) | 0.185 |  | 32(3.9) | 14(4.4) | 0.726 |  | 660(25.5) | 357(22.1) | 0.001 |
| Cerebrovascular disease | 84(7.6) | 67(7.3) | 0.78 |  | 33(4.0) | 14(4.4) | 0.800 |  | 240(5.6) | 120(4.5) | 0.036 |
| Chronic heart disease | 48(4.4) | 38(4.1) | 0.809 |  | 10(1.2) | 4(1.2) | 0.972 |  | 125(2.9) | 75(2.8) | 0.751 |
| Others | 19(1.7) | 25(2.7) | 0.126 |  | 19(2.3) | 6(1.9) | 0.64 |  | 59(1.4) | 38(1.4) | 0.903 |
| **Infectious diseases** | 136(12.4) | 86(9.4) | 0.033 |  | 27(3.3) | 17(5.3) | 0.115 |  | 408(9.6) | 291(10.8) | 0.109 |
| Pneumonia or sepsis | 84(7.6) | 52(5.7) | 0.08 |  | 18(2.2) | 12(3.7) | 0.144 |  | 257(6.0) | 179(6.7) | 0.272 |
| Aspergillosis | 34(3.1) | 24(2.6) | 0.527 |  | 5(0.6) | 3(0.9) | 0.845 |  | 110(2.6) | 77(2.9) | 0.459 |
| Tuberculosis | 8(0.4) | 4(0.7) | 0.397 |  | 2(0.2) | 1(0.3) | 1.000 |  | 24(0.6) | 17(0.6) | 0.703 |
| Others | 10(0.9) | 6(0.7) | 0.521 |  | 2(0.2) | 1(0.3) | 1.000 |  | 17(0.4) | 18(0.7) | 0.117 |
| **Respiratory diseases** | 47(4.3) | 32(3.4) | 0.301 |  | 16(2.0) | 6(1.9) | 0.926 |  | 361(8.5) | 137(5.1) | <0.001 |
| Chronic obstructive pulmonary diseases | 30(2.7) | 20(2.2) | 0.432 |  | 8(1.0) | 3(0.9) | 0.948 |  | 236(5.6) | 89(3.3) | <0.001 |
| Pulmonary arterial hypertension | 9(0.8) | 7(0.8) | 0.89 |  | 4(0.5) | 1(0.3) | 1.000 |  | 75(1.8) | 29(1.1) | 0.024 |
| Lung fibrosis | 3(0.3) | 2(0.2) | 0.806 |  | 2(0.2) | 1(0.3) | 1.000 |  | 20(0.5) | 7(0.3) | 0.176 |
| Others | 5(0.5) | 3(0.3) | 0.651 |  | 2(0.2) | 1(0.3) | 1.000 |  | 30(0.7) | 12(0.4) | 0.181 |
| **Digestive diseases** | 35(3.2) | 35(3.8) | 0.437 |  | 14(1.7) | 16(5.0) | <0.001 |  | 157(3.7) | 71(2.7) | 0.019 |
| Pancreatitis | 18(1.6) | 20(2.2) | 0.37 |  | 7(0.9) | 8(2.5) | 0.025 |  | 86(2.0) | 38(1.4) | 0.067 |
| Upper gastrointestinal hemorrhage | 13(1.2) | 11(1.2) | 0.97 |  | 5(0.6) | 6(1.9) | 0.106 |  | 58(1.4) | 25(0.9) | 0.111 |
| Others | 4(0.4) | 4(0.4) | 0.796 |  | 2(0.2) | 2(0.6) | 0.677 |  | 13(0.3) | 8(0.3) | 0.964 |
| **Renal diseases** | 77(7.1) | 74(8.0) | 0.406 |  | 38(4.6) | 3(0.9) | 0.003 |  | 263(6.2) | 124(4.7) | 0.008 |
| Chronic kidney diseases | 38(3.5) | 40(4.4) | 0.347 |  | 21(2.6) | 2(0.6) | 0.036 |  | 146(3.4) | 68(2.5) | 0.038 |
| Acute kidney injury | 31(2.8) | 28(3.1) | 0.754 |  | 15(1.8) | 0(0) | 0.031 |  | 94(2.2) | 44(1.6) | 0.103 |
| Others | 8(0.7) | 6(0.7) | 0.844 |  | 2(0.2) | 1(0.3) | 0.842 |  | 23(0.5) | 12(0.4) | 0.601 |
| **Endocrine diseases** | 23(2.1) | 47(5.1) | <0.001 |  | 0(0) | 3(0.9) | 0.006 |  | 76(1.8) | 83(3.3) | <0.001 |
| Diabetes mellitus | 16(1.5) | 30(3.3) | 0.006 |  | 0(0) | 2(0.6) | 0.14 |  | 46(1.1) | 51(1.9) | 0.004 |
| Thyrotoxicosis | 5(0.5) | 10(1.1) | 0.098 |  | 0(0) | 1(0.3) | 0.627 |  | 20(0.5) | 21(0.8) | 0.095 |
| Others | 2(0.2) | 7(0.8) | 0.051 |  | 0(0) | 0(0) | - |  | 10(0.2) | 11(0.4) | 0.193 |
| **Others** | 60(5.5) | 86(9.4) | <0.001 |  | 23(2.8) | 14(4.4) | 0.183 |  | 544(12.8) | 486(17.9) | <0.001 |

Chi-squared test for the cause of death between male and female in three groups.

Supplementary table 3. Cause of deaths in patients with NAFLD and non-NAFLD, stratified by BMI

| Diseases, n (%) | NAFLD (n=2015) | | |  | Other liver diseases(n=1140) | | |  | No liver diseases  (n=6916) | | |
| --- | --- | --- | --- | --- | --- | --- | --- | --- | --- | --- | --- |
|  | BMI<25  (n=1067) | BMI≥25  (n=948) | *P* |  | BMI<25  (n=877) | BMI≥25  (n=263) | *P* |  | BMI<25  (n=5325) | BMI≥25  (n=1591) | *P* |
| **Liver-related diseases** | 62(5.8) | 43(4.5) | 0.199 |  | 523(59.6) | 142(54) | 0.104 |  | 125(2.3) | 34(2.1) | 0.623 |
| Hepatocellular carcinoma | 29(2.7) | 21(2.2) | 0.469 |  | 257(29.3) | 70(26.6) | 0.398 |  | 82(1.5) | 22(1.4) | 0.651 |
| Decompensated cirrhosis | 13(1.2) | 10(1.1) | 0.730 |  | 170(19.4) | 46(17.5) | 0.492 |  | 16(0.3) | 5(0.3) | 1.000 |
| Hepatic failure | 20(1.9) | 12(1.3) | 0.275 |  | 96(10.9) | 26(9.9) | 0.626 |  | 27(0.5) | 7(0.4) | 0.737 |
| **Extrahepatic neoplasms** | 240(22.5) | 215(22.6) | 0.92 |  | 129(14.7) | 37(14.1) | 0.796 |  | 1601(30.1) | 481(30.2) | 0.899 |
| Colorectal cancer | 67(6.3) | 60(6.3) | 0.963 |  | 36(4.1) | 9(3.4) | 0.618 |  | 280(5.3) | 66(4.1) | 0.075 |
| Lung cancer | 33(3.1) | 29(3.1) | 0.965 |  | 23(2.6) | 7(2.7) | 0.972 |  | 270(5.1) | 90(5.7) | 0.356 |
| Stomach cancer | 24(2.2) | 23(2.3) | 0.793 |  | 20(2.3) | 5(1.9) | 0.713 |  | 212(4.0) | 72(4.5) | 0.337 |
| Hematologic malignancy | 53(5.0) | 46(4.9) | 0.905 |  | 31(3.5) | 10(3.8) | 0.838 |  | 315(5.9) | 107(6.7) | 0.236 |
| Other malignancies | 63(5.9) | 57(6.0) | 0.918 |  | 19(2.2) | 6(2.3) | 0.911 |  | 524(9.8) | 146(9.2) | 0.432 |
| **Cardio- and cerebrovascular diseases** | 323(30.3) | 394(35.6) | <0.001 |  | 89(10.1) | 43(16.3) | 0.006 |  | 1208(22.7) | 466(29.3) | <0.001 |
| Ischemic heart disease | 197(18.5) | 240(21.7) | <0.001 |  | 31(3.5) | 15(5.7) | 0.117 |  | 734(13.8) | 283(17.8) | <0.001 |
| Cerebrovascular disease | 68(6.4) | 83(7.5) | 0.043 |  | 31(3.5) | 16(6.1) | 0.068 |  | 260(4.9) | 100(6.3) | 0.027 |
| Chronic heart disease | 36(3.4) | 50(4.3) | 0.035 |  | 9(1.0) | 5(1.9) | 0.258 |  | 144(2.7) | 56(3.5) | 0.088 |
| Others | 22(2.1) | 21(2.1) | 0.812 |  | 18(2.1) | 7(2.7) | 0.554 |  | 70(1.3) | 27(1.7) | 0.255 |
| **Infectious diseases** | 143(13.4) | 78(11.1) | <0.001 |  | 36(4.1) | 8(3.0) | 0.432 |  | 562(10.6) | 137(8.6) | 0.024 |
| Pneumonia or sepsis | 89(8.3) | 48(6.8) | 0.004 |  | 25(2.9) | 5(1.9) | 0.399 |  | 350(6.6) | 86(5.4) | 0.093 |
| Aspergillosis | 37(3.5) | 20(2.8) | 0.066 |  | 7(0.8) | 1(0.4) | 0.771 |  | 150(2.8) | 27(1.7) | 0.013 |
| Tuberculosis | 8(0.7) | 4(0.6) | 0.340 |  | 2(0.2） | 1(0.4) | 1.000 |  | 33(0.6) | 8(0.5) | 0.594 |
| Others | 9(0.8) | 6(0.7) | 0.583 |  | 2(0.2） | 1(0.4) | 1.000 |  | 29(0.5) | 6(0.4) | 0.409 |
| **Respiratory diseases** | 45(4.2) | 34(3.9) | 0.466 |  | 17(1.9) | 5(1.9) | 0.969 |  | 403(7.6) | 95(6.0) | 0.031 |
| Chronic obstructive pulmonary diseases | 28(2.6) | 22(2.5) | 0.662 |  | 8(0.9) | 3(1.1) | 0.740 |  | 263(4.9) | 62(3.9) | 0.085 |
| Pulmonary arterial hypertension | 9(0.8) | 7(0.8) | 0.791 |  | 4(0.5) | 1(0.4) | 0.870 |  | 84(1.6) | 20(1.3) | 0.357 |
| Lung fibrosis | 2(0.2) | 2(0.2) | 0.906 |  | 2(0.2） | 1(0.4) | 1.000 |  | 22(0.4) | 5(0.3) | 0.579 |
| Others | 6(0.6) | 5(0.5) | 0.915 |  | 3(0.3) | 0(0) | 0.792 |  | 34(0.6) | 8(0.5) | 0.541 |
| **Digestive diseases** | 39(3.7) | 31(3.5) | 0.638 |  | 23(2.6) | 7(2.7) | 0.972 |  | 185(3.5) | 43(2.7) | 0.130 |
| Pancreatitis | 21(2.0) | 17(1.9) | 0.773 |  | 12(1.4) | 3(1.1) | 1.000 |  | 100(1.9) | 24(1.5) | 0.330 |
| Upper gastrointestinal hemorrhage | 13(1.2) | 11(1.2) | 0.905 |  | 8(0.9) | 3(1.1) | 1.000 |  | 68(1.3) | 15(0.9) | 0.283 |
| Others | 5(0.5) | 3(0.3) | 0.851 |  | 3(0.3) | 1(0.4) | 1.000 |  | 17(0.3) | 4(0.3) | 0.666 |
| **Renal diseases** | 93(8.7) | 58(6.1) | 0.027 |  | 32(3.6) | 9(3.2) | 0.862 |  | 305(5.7) | 82(5.2) | 0.382 |
| [Chronic](D:/Program%20Files%20(x86)/Youdao/Dict/8.5.3.0/resultui/html/index.html#/javascript:;) [kidney](D:/Program%20Files%20(x86)/Youdao/Dict/8.5.3.0/resultui/html/index.html#/javascript:;) [diseases](D:/Program%20Files%20(x86)/Youdao/Dict/8.5.3.0/resultui/html/index.html#/javascript:;) | 49(4.6) | 30(3.2) | 0.099 |  | 18(2.1) | 5(1.9) | 0.878 |  | 169(3.2) | 45(2.8) | 0.485 |
| Acute kidney injury | 36(3.4) | 22(2.3) | 0.158 |  | 12(1.4) | 3(1.1) | 1.000 |  | 109(2.0) | 29(1.8) | 0.575 |
| Others | 8(0.7) | 6(0.7) | 0.753 |  | 2(0.2） | 1(0.4) | 1.000 |  | 27(0.5) | 8(0.5) | 0.983 |
| **Endocrine diseases** | 30(2.9) | 40(4.2) | 0.110 |  | 1(0.1) | 2(0.8) | 0.268 |  | 111(2.1) | 48(3.0) | 0.029 |
| [Diabetes](D:/Program%20Files%20(x86)/Youdao/Dict/8.5.3.0/resultui/html/index.html#/javascript:;) [mellitus](D:/Program%20Files%20(x86)/Youdao/Dict/8.5.3.0/resultui/html/index.html#/javascript:;) | 20(1.9) | 26(2.7) | 0.193 |  | 1(0.1) | 1(0.4) | 0.948 |  | 68(1.3) | 29(1.8) | 0.104 |
| Thyrotoxicosis | 5(0.5) | 7(0.6) | 0.432 |  | 0(0) | 1(0.4) | 0.522 |  | 28(0.5) | 13(0.8) | 0.184 |
| Others | 5(0.6) | 7(0.7) | 0.622 |  | 0(0) | 0(0) | - |  | 15(0.3) | 6(0.4) | 0.544 |
| **Others** | 91(8.4) | 55(5.8) | 0.022 |  | 27(3.1) | 10(3.8) | 0.561 |  | 825(15.5) | 205(12.9) | 0.010 |

Abbreviations: BMI, body mass index.

Chi-squared test for the cause of death between BMI<25 and BMI≥25 in three groups

Supplementary table 4. Cause of deaths in patients with NAFLD, stratified by FIB-4

| Diseases, n(%) | FIB-4 < 1.3  (n=1642) | FIB-4 ≥ 1.3  (n=373) | *P* |
| --- | --- | --- | --- |
| **Liver-related diseases**  diseases | 67(4.1) | 38(10.2) | <0.001 |
| Hepatocellular carcinoma | 32(1.9) | 19(5.1) | <0.001 |
| Decompensated cirrhosis | 14(0.9) | 8(2.1) | 0.030 |
| Hepatic failure | 21(1.3) | 11(2.9) | 0.020 |
| **Extrahepatic neoplasms** | 356(21.7) | 99(26.5) | 0.043 |
| Colorectal cancer | 93(5.7) | 32(8.6) | 0.035 |
| Lung cancer | 51(3.1) | 11(2.9) | 0.874 |
| Stomach cancer | 40(2.4) | 10(2.7) | 0.784 |
| Hematologic malignancy | 78(4.8) | 19(5.1) | 0.780 |
| Other malignancies | 94(5.7) | 27(7.2) | 0.267 |
| **Cardio- and cerebro- vascular diseases** | 567(34.5) | 150(40.2) | 0.038 |
| Ischemic heart disease | 341(20.8) | 95(25.5) | 0.047 |
| Cerebrovascular disease | 121(7.4) | 30(8.0) | 0.655 |
| Chronic heart disease | 71(4.3) | 15(4.0) | 0.794 |
| Others | 34(2.1) | 10(2.7) | 0.467 |
| **Infectious diseases** | 195(11.9) | 27(7.2) | 0.010 |
| Pneumonia or sepsis | 120(7.3) | 16(4.3) | 0.036 |
| Aspergillosis | 51(3.1) | 7(1.9) | 0.200 |
| Tuberculosis | 10(0.6) | 2(0.5) | 1.000 |
| Others | 14(0.9) | 2(0.5) | 0.765 |
| **Respiratory diseases** | 70(4.3) | 9(2.4) | 0.097 |
| Chronic obstructive pulmonary diseases | 45(2.7) | 5(1.3) | 0.117 |
| Pulmonary arterial hypertension | 14(0.9) | 2(0.5) | 0.534 |
| Lung fibrosis | 4(0.2) | 1(0.3) | 1.000 |
| Others | 7(0.4) | 1(0.3) | 1.000 |
| **Digestive diseases** | 62(3.8) | 8(2.1) | 0.120 |
| Pancreatitis | 34(2.1) | 4(1.1) | 0.201 |
| Upper gastrointestinal hemorrhage | 21(1.3) | 3(0.8) | 0.446 |
| Others | 7(0.4) | 1(0.3) | 1.000 |
| **Renal diseases** | 132(8.0) | 19(5.1) | 0.051 |
| Chronic kidney diseases | 69(4.2) | 9(2.4) | 0.106 |
| Acute kidney injury | 51(3.1) | 8(2.1) | 0.320 |
| Others | 12(0.7) | 2(0.5) | 0.683 |
| **Endocrine diseases** | 61(3.7) | 9(2.4) | 0.215 |
| Diabetes mellitus | 40(2.4) | 6(1.6) | 0.334 |
| Thyrotoxicosis | 12(0.7) | 3(0.8) | 0.882 |
| Others | 8(0.5) | 1(0.3) | 0.886 |
| **Others** | 132(8.0) | 14(3.8) | 0.004 |

Abbreviations: FIB-4, fibrosis index based on the 4 factor

Chi-squared test for the cause of death between FIB-4 < 1.3 and FIB-4 ≥ 1.3 in NAFLD group.

Supplementary table 5. Baseline characteristics of the deaths with NAFLD and non-NAFLD with ascribing cryptogenic cirrhosis to Other liver diseases group.

| Characteristics | NAFLD  (n=1972) | Other liver diseases  (n=1183) | No liver diseases  (n=6916) | *P* | Post-hoc | | |
| --- | --- | --- | --- | --- | --- | --- | --- |
|  |  |  |  |  | NA vs. OL | NA vs. NL | OL vs. NL |
| Male, n (%) | 1074(54.5) | 844(71.3) | 4248(61.4) | <0.001 | <0.001 | 0.023 | 0.002 |
| Age, year | 64.2±18.8 | 57.6±16.3 | 66.1±19.2 | <0.001 | <0.001 | 0.341 | <0.001 |
| BMI, kg/m^2^ | 24.3±5.5 | 21.4±3.2 | 21.6±3.6 | <0.001 | <0.001 | <0.001 | 0.410 |
| SBP, mmHg | 133.7±23.2 | 125.6±24.7 | 128.7±25.4 | 0.004 | <0.001 | 0.026 | 0.056 |
| DBP, mmHg | 79.5±14.3 | 75.2±15.5 | 75.1±13.5 | 0.070 | - | - | - |
| ALT, U/L^a^ | 32(16-50) | 50(25-81) | 31(17-53) | <0.001 | <0.001 | 0.450 | <0.001 |
| AST,U/L^a^ | 45(24-71) | 106(69-166) | 49(25-83) | <0.001 | <0.001 | 0.562 | <0.001 |
| GGT, U/L^a^ | 62(31-104) | 63(32-102) | 53(26-96) | <0.001 | 0.850 | 0.002 | 0.007 |
| ALP, U/L^a^ | 93(66-141) | 113(76-171) | 94(66-121) | <0.001 | 0.001 | 0.630 | <0.001 |
| Total cholesterol, mmol/L | 5.24±1.28 | 2.71±1.23 | 3.53±1.17 | <0.001 | <0.001 | <0.001 | <0.001 |
| Triglycerides, mmol/L | 2.84±1.44 | 1.00±0.46 | 1.11±0.48 | <0.001 | <0.001 | <0.001 | 0.960 |
| HDL-cholesterol, mmol/L | 0.94±0.32 | 0.64±0.25 | 0.88±0.23 | <0.001 | <0.001 | 0.004 | <0.001 |
| LDL-cholesterol, mmol/L | 3.26±1.55 | 1.70±0.88 | 2.21±0.86 | <0.001 | <0.001 | <0.001 | <0.001 |
| Apolipoprotein-A1, g/L | 0.64±0.35 | 1.00±0.38 | 0.90±0.30 | <0.001 | <0.001 | <0.001 | <0.001 |
| Apolipoprotein-B, g/L | 0.98±0.36 | 0.58±0.25 | 0.71±0.22 | <0.001 | <0.001 | <0.001 | <0.001 |
| Lipoprotein-A, mg/L | 350.3±41.2 | 162.1±23.5 | 289.3±32.3 | <0.001 | <0.001 | 0.005 | 0.001 |
| FBG, mmol/L | 7.9±2.3 | 6.4±1.5 | 7.1±2.0 | <0.001 | <0.001 | <0.001 | 0.023 |
| Uric acid, umol/L | 369.5±40.5 | 304.1±30.3 | 334.4±32.4 | <0.001 | <0.001 | 0.034 | 0.086 |

Abbreviations: BMI, body mass index; SBP, systolic blood pressure; DBP, diastolic blood pressure; ALT, alanine aminotransferase; AST, aspartate aminotransferase; GGT, gamma glutamyl transpeptidase; ALP, alkaline phosphatase; HDL-cholesterol, high-density lipoprotein-cholesterol; LDL-cholesterol, low-density lipoprotein-cholesterol; FBG, fasting blood glucose. ^a^ Continuous variables are expressed as median with 25^th^-75^th^ interquartile range for non-Gaussian distribution.

Supplementary table 6. Cause of deaths in patients with NAFLD vs non-NAFLD with ascribing cryptogenic cirrhosis to Other liver diseases group.

| Diseases, n (%) | NAFLD  (n=1972) | Other liver diseases  (n=1183) | No liver diseases  (n=6916) | *P* | Post-hoc | | |
| --- | --- | --- | --- | --- | --- | --- | --- |
|  |  |  |  |  | NA vs. OL | NA vs. NL | OL vs. NL |
| **Liver-related diseases** | 93(4.7) | 677(57.2) | 159(2.3) | <0.001 | <0.001 | <0.001 | <0.001 |
| Hepatocellular carcinoma | 47(2.4) | 331(28.0) | 104(1.5) | <0.001 | <0.001 | 0.004 | <0.001 |
| Decompensated cirrhosis | 18(0.9) | 220(18.6) | 21(0.3) | <0.001 | <0.001 | <0.001 | <0.001 |
| Hepatic failure | 28(1.4) | 126(10.7) | 34(0.5) | <0.001 | <0.001 | <0.001 | <0.001 |
| **Extrahepatic neoplasms** | 440(22.3) | 181(15.3) | 2082(30.1) | <0.001 | <0.001 | <0.001 | <0.001 |
| Colorectal cancer | 122(6.2) | 48(4.1) | 346(5.0) | 0.012 | 0.005 | 0.023 | 0.250 |
| Lung cancer | 62(3.1) | 33(2.8) | 360(5.2) | <0.001 | 0.520 | <0.001 | <0.001 |
| Stomach cancer | 47(2.4) | 28(2.4) | 284(4.1) | <0.001 | 0.750 | 0.001 | 0.009 |
| Hematologic malignancy | 94(4.8) | 44(3.7) | 422(6.1) | 0.001 | 0.330 | 0.080 | 0.002 |
| Other malignancies | 115(5.8) | 28(2.4) | 670(9.7) | <0.001 | <0.001 | <0.001 | <0.001 |
| **Cardio- and cerebrovascular diseases** | 713(36.1) | 136(11.5) | 1674(24.2) | <0.001 | <0.001 | <0.001 | <0.001 |
| Ischemic heart disease | 434(22.0) | 48(4.1) | 1017(14.7) | <0.001 | <0.001 | <0.001 | <0.001 |
| Cerebrovascular disease | 150(7.6) | 48(4.1) | 360(5.2) | <0.001 | <0.001 | <0.001 | 0.365 |
| Chronic heart disease | 85(4.3) | 15(1.3) | 200(2.9) | <0.001 | <0.001 | 0.002 | 0.002 |
| Others | 44(2.2) | 25(2.1) | 97(1.4) | 0.024 | 0.920 | 0.028 | 0.062 |
| **Infectious diseases** | 219(11.1) | 47(4.0) | 699(10.1) | <0.001 | <0.001 | 0.560 | <0.001 |
| Pneumonia or sepsis | 135(6.8) | 31(2.6) | 436(6.3) | <0.001 | <0.001 | 0.860 | <0.001 |
| Aspergillosis | 57(2.9) | 9(0.8) | 187(2.7) | <0.001 | <0.001 | 0.620 | <0.001 |
| Tuberculosis | 11(0.6) | 4(0.3) | 41(0.6) | 0.513 | - | - | - |
| Others | 16(0.8) | 3(0.3) | 35(0.5) | 0.145 | - | - | - |
| **Respiratory diseases** | 76(3.9) | 25(2.1) | 498(7.2) | <0.001 | 0.004 | <0.001 | <0.00 |
| Chronic obstructive pulmonary diseases | 49(2.5) | 11(0.9) | 325(4.7) | <0.001 | 0.004 | <0.001 | <0.001 |
| Pulmonary arterial hypertension | 15(0.8) | 6(0.5) | 104(1.5) | 0.003 | 0.520 | 0.040 | 0.030 |
| Lung fibrosis | 4(0.2) | 4(0.3) | 27(0.4) | 0.650 | - | - | - |
| Others | 8(0.4) | 4(0.3) | 42(0.6) | 0.260 | - | - | - |
| **Digestive diseases** | 68(3.4) | 32(2.7) | 228(3.3) | 0.560 | - | - | - |
| Pancreatitis | 37(1.9) | 16(1.4) | 124(1.8) | 0.620 | - | - | - |
| Upper gastrointestinal hemorrhage | 23(1.2) | 12(1.0) | 83(1.2) | 0.860 | - | - | - |
| Others | 8(0.4) | 4(0.3) | 21(0.3) | 0.830 | - | - | - |
| **Renal diseases** | 149(7.6) | 43(3.6) | 387(5.6) | <0.001 | <0.001 | 0.006 | 0.040 |
| [Chronic](D:/Program%20Files%20(x86)/Youdao/Dict/8.5.3.0/resultui/html/index.html#/javascript:;) [kidney](D:/Program%20Files%20(x86)/Youdao/Dict/8.5.3.0/resultui/html/index.html#/javascript:;) [diseases](D:/Program%20Files%20(x86)/Youdao/Dict/8.5.3.0/resultui/html/index.html#/javascript:;) | 77(3.9) | 24(2.0) | 214(3.1) | 0.024 | 0.022 | 0.190 | 0.150 |
| Acute kidney injury | 58(2.9) | 16(1.4) | 138(2.0) | 0.009 | 0.014 | 0.026 | 0.320 |
| Others | 14(0.7) | 3(0.3) | 35(0.5) | 0.310 | - | - | - |
| **Endocrine diseases** | 68(3.4) | 5(0.4) | 159(2.3) | <0.001 | <0.001 | 0.008 | <0.001 |
| [Diabetes](D:/Program%20Files%20(x86)/Youdao/Dict/8.5.3.0/resultui/html/index.html#/javascript:;) [mellitus](D:/Program%20Files%20(x86)/Youdao/Dict/8.5.3.0/resultui/html/index.html#/javascript:;) | 45(2.3) | 3(0.3) | 97(1.4) | <0.001 | <0.001 | 0.024 | 0.004 |
| Thyrotoxicosis | 14(0.7) | 2(0.2) | 41(0.6) | 0.080 | - | - | - |
| Others | 9(0.5) | 0(0) | 21(0.3) | 0.092 | - | - | - |
| **Others** | 146(7.4) | 37(3.1) | 1030(14.8) | <0.001 | <0.001 | <0.001 | <0.001 |

Abbreviations: NA, NAFLD; OL, Other liver diseases; NL, No liver diseases.

Chi-squared test for the comparison of cause of deaths distribution between NAFLD and non-NAFLD.

Supplementary table 7. Association between steatosis degrees and distribution of causes of death with ascribing cryptogenic cirrhosis to Other liver diseases Non-NAFLD group.

| Diseases, n (%) | Non-NAFLD  (n=6916) | Mild  (n=522) | Moderate  (n=1102) | Severe  (n=348) | Univariate | | Multivariate | |
| --- | --- | --- | --- | --- | --- | --- | --- | --- |
|  |  |  |  |  | OR (95%CI) | *P* | OR (95%CI) | *P* |
| Liver-related diseases | 159(2.3) | 12(2.3) | 61(5.5) | 20(5.7) | 1.23(1.05-1.35) | <0.001 | 1.22(1.03-1.44) | <0.001 |
| Extrahepatic neoplasms | 2082(30.1) | 167(32.0) | 228(20.7) | 45(12.9) | 0.93(0.86-0.99) | 0.030 | 0.97(0.84-1.03) | 0.230 |
| Cardio- and cerebrovascular  diseases | 1674(24.2) | 205(39.3) | 394(35.8) | 114(32.8) | 1.21(1.14-1.28) | <0.001 | 1.25(1.20-1.32) | <0.001 |
| Infectious diseases | 699(10.1) | 53(10.2) | 104(9.4) | 62(17.8) | 1.15(1.01-1.28) | 0.025 | 1.16(1.02-1.32) | 0.036 |
| Respiratory diseases | 498(7.2) | 25(4.8) | 40(3.6) | 11(3.2) | 0.88(0.77-0.98) | 0.010 | 0.89(0.78-1.11) | 0.280 |
| Digestive diseases | 228(3.3) | 16(3.1) | 35(3.2) | 17(4.9) | 1.04(0.81-1.39) | 0.880 | 1.06(0.81-1.39) | 0.760 |
| Renal diseases | 387(5.6) | 16(3.1) | 110(10.0) | 23(6.6) | 1.09(0.99-1.38) | 0.067 | 1.10(0.98-1.41) | 0.078 |
| Endocrine diseases | 159(2.3) | 9(1.7) | 43(3.9) | 16(4.6) | 1.18(0.92-1.61) | 0.560 | 1.17(0.90-1.59) | 0.450 |
| Others | 1030(14.9) | 19(3.6) | 87(7.9) | 40(11.5) | 0.88(0.78-1.02) | 0.052 | 0.89(0.79-1.02) | 0.051 |

Multivariate logistics regression analysis adjusted for age, sex, body mass index, metabolic syndrome and FIB-4.

Abbreviations: FIB-4, fibrosis index based on the 4 factor; FIB-4=age(year)*AST(U/L)/PLT(10^9^/L)*ALT(U/L)^0.5

Supplementary table 8. Anthropometrical and metabolic characteristics of different causes of deaths with NAFLD without including cryptogenic cirrhosis.

| Characteristics | Liver-related diseases  (n=93) | Extrahepatic neoplasms  (n=440) | Cardio-and cerebrovascular  diseases  (n=713) | Infectious diseases  (n=219) | Respiratory diseases  (n=76) | Digestive diseases  (n=68) | Renal Diseases  (n=149) | Endocrine diseases  (n=68) | Others  (n=146) | *P* |
| --- | --- | --- | --- | --- | --- | --- | --- | --- | --- | --- |
| Male, n (%) | 72(82.8)  bcdefgh | 230(52.3)  ahi | 399(56.0)  ahi | 134(61.2)  aghi | 45(59.2)  ahi | 34(50.0)  adhi | 78(52.3)  adhi | 22(32.4)  abcdefg | 56(38.4)  abcdefg | <0.001 |
| Age, year | 54.1±17.7  bcdefgh | 65.6±16.3  aghi | 70.1±15.7  agi | 66.5±19.2  agi | 65.2±18.5  aghi | 64.6±19.5  aghi | 59.3±20.9  abcdefh | 71.1±14.1  abefgi | 56.7±20.2  bcdefh | <0.001 |
| BMI, kg/m^2^ | 27.2±3.8  bg | 23.0±4.2  afh | 24.5±4.8  gh | 24.8±6.3  gh | 25.4±7.9  gh | 25.9±5.0  bgh | 21.3±3.3  acdefhi | 30.5±8.2  bcdefgi | 24.5±4.7  gh | 0.042 |
| SBP, mmHg | 131.5±16.0  cd | 129.6±10.5  eghi | 150.3±21.8  abdefhi | 130.6±10.4  bcfgh | 137.4±19.0  abcfgh | 131.6±11.9  cdegh | 149.8 ±23.6  abdefhi | 158.0±18.1  abcdefgi | 134.0±10.8  cgh | <0.001 |
| DBP, mmHg | 81.6±8.2  gh | 76.9±7.2  cefgh | 86.8±9.8  bdi | 76.7±8.9  cfgh | 79.9±7.3  bgh | 83.8±9.6  bdghi | 89.8±9.2  abdefi | 89.7±9.9  abdefi | 78.8±8.1  cfgh | <0.001 |
| ALT, U/L^†^ | 56(33-65)  bcdefghi | 30(16-43)  adg | 30(16-46)  adg | 42(17-65)  abcefghi | 28(16-38)  adg | 24(14-40)  adghi | 21(9-28)  abcdefhi | 33(11-50)  adfg | 33(15-50)  adfg | <0.001 |
| AST, U/L^†^ | 114(67-160)  bcdefghi | 50(24-73)  acdefg | 39(23-53)  abdhi | 61(28-88)  abcefghi | 38(24-50)  abdhi | 34(22-44)  abdhi | 31(19-45)  abdhi | 50(24-80)  acdefg | 48(25-72)  acdefg | <0.001 |
| GGT, U/L^†^ | 103(35-158) | 74(31-110) | 57(30-78) | 75(35-105) | 55(32-75) | 53(26-75) | 44(30-55) | 54(22-78) | 70(30-89) | 0.380 |
| ALP, U/L^†^ | 190(113-255)  bcdefghi | 115(68-154)  acdeghi | 84(63-101)  abdefi | 132(83-167)  abcefghi | 91(68-110)  abcdfg | 113(67-150)  acdeghi | 76(53-100)  abdefhi | 86(63-105)  abdfg | 94(65-118)  abcdfg | <0.001 |
| Total cholesterol, mmol/L | 5.7±1.4  defi | 5.7±1.4  defi | 5.5±1.0  dei | 4.3±1.0  abcefghi | 5.0±0.9  abcdfgh | 3.7±1.2  abdeghi | 5.5±1.1  defi | 5.5±1.5  defi | 4.8±1.3  abcdfgh | 0.001 |
| Triglycerides, mmol/L | 2.5±1.5  bdefgi | 2.7±1.0  acdhi | 2.5±1.2  bdefgi | 3.8±1.2  abcefh | 2.8±1.0  acdhi | 3.0±1.1  acdhi | 3.4±1.5  ach | 2.4±1.4  bdefgi | 3.8±2.4  abcefh | 0.008 |
| HDL-cholesterol, mmol/L | 0.72±0.16  bceg | 1.05±0.25  adfi | 1.09±0.22  adfi | 0.76±0.14  bcef | 1.23±0.22  adfi | 0.55±0.15  bcdeghi | 1.00±0.21  afi | 1.04±0.22  dfi | 0.75±0.12  bcefgh | <0.001 |
| LDL-cholesterol, mmol/L | 3.64±1.19  dfi | 3.58±1.29  dfi | 3.49±1.09  dfi | 2.47±1.1  abcegh | 3.52±1.17  dfi | 2.26±0.52  abcegh | 3.25±1.61  df | 3.52±1.14  dfi | 2.85±0.64  abceh | <0.001 |
| Apolipoprotein-A1, g/L | 0.66±0.36  cegh | 0.98±0.51  cef | 1.17±0.44 | 0.80±0.45 | 1.25±0.48 | 0.61±0.24 | 1.09±0.44 | 1.14±0.35 | 0.82±0.43 | <0.001 |
| Apolipoprotein-B, g/L | 0.90±0.39 | 1.02±0.35 | 0.99±0.34 | 0.86±0.28 | 0.97±0.24 | 0.68±0.40 | 0.96±0.24 | 1.05±0.26 | 1.06±0.25 | 0.220 |
| Lipoprotein-A, mg/L | 236.8±36.5  bcdeghi | 258.9±39.9  acefghi | 440.2±60.0  abdefi | 264.1±63.7  acefghi | 329.2±53.5  abcdfgh | 207.6±64.1  bcdeghi | 440.3±33.2  abdefi | 434.1±46.2  abdefi | 326.3±63.1  abcdfgh | 0.049 |
| FBG, mmol/L | 7.5±1.6 | 8.2±1.7 | 8.6±1.8 | 8.5±2.2 | 7.7±1.0 | 8.0±1.8 | 7.3±1.5 | 9.1±2.5 | 7.7±2.1 | 0.280 |
| Uric acid, umol/L | 378.0±39.2 | 385.3±37.3 | 411.6±38.8 | 384.9.0±33.4 | 443.5±38.7 | 389.2±38.3 | 412.5±50.5 | 339.0±38.3 | 362.9±38.5 | 0.290 |

Abbreviations: BMI, body mass index; SBP, systolic blood pressure; DBP, diastolic blood pressure; ALT, alanine aminotransferase; AST, aspartate aminotransferase; GGT, gamma glutamyl transpeptidase; ALP, alkaline phosphatase; HDL-cholesterol, high-density lipoprotein-cholesterol; LDL-cholesterol, low-density lipoprotein-cholesterol; FBG, fasting blood glucose;

^†^Continuous variables are expressed as median with IQR for non-Gaussian distribution. *P* values were for the ANOVA analysis across the groups, different. a, b, c, d, e, f, g, h, i- refer to statistic significant after post-hoc multiple comparisons with Bonferroni adjustments when compared with Liver-related diseases group(a), Extrahepatic neoplasms group(b), Cardio-and cerebrovascular diseases group(c), Infectious diseases group(d), Respiratory diseases group(e), Digestive diseases group(f), Renal diseases group(g), Endocrine diseases group(h) and Others(i).

Supplementary table 9. The occurrence of multiple organ dysfunction syndrome (MODS) in patients with NAFLD vs. non-NAFLD with ascribing cryptogenic cirrhosis to Other liver diseases group.

| Organ dysfunction, n(%) | NAFLD  (n=1972) | Other liver diseases  (n=1183) | No liver diseases  (n=6916) | *P* | Post-hoc | | |
| --- | --- | --- | --- | --- | --- | --- | --- |
|  |  |  |  |  | NA vs. OL | NA vs. NL | OL vs. NL |
| Heart failure | 838(42.5) | 415(35.1) | 2185(31.6) | <0.001 | <0.001 | <0.001 | 0.006 |
| Respiratory failure | 872(44.2) | 408(34.5) | 2248(32.5) | <0.001 | <0.001 | <0.001 | 0.102 |
| Renal failure | 647(32.8) | 271(22.9) | 1480(21.4) | <0.001 | <0.001 | <0.001 | 1.000 |
| Hepatic failure | 266(13.5) | 318(26.9) | 539(7.8) | <0.001 | <0.001 | <0.001 | <0.001 |
| Disseminated intravascular coagulation | 245(12.4) | 129(10.9) | 387(5.6) | <0.001 | 0.345 | <0.001 | <0.001 |
| Two organ dysfunctions | 303 (15.4) | 137(11.3) | 657(9.5) | <0.001 | 0.004 | <0.001 | 0.100 |
| MODS | 603(30.6) | 300(25.4) | 1494(21.6) | <0.001 | 0.049 | <0.001 | 0.012 |

Abbreviations: NA, NAFLD; OL, Other liver diseases; NL, No liver diseases; MODS, multiple organ dysfunction syndrome.

Chi-squared test for the occurrence of organ dysfunction, which included single, two and multiple organ dysfunction between NAFLD and non-NAFLD.

Supplementary table 10. Cause of deaths in patients with NAFLD and non-NAFLD with ascribing cryptogenic cirrhosis to Other liver diseases group, stratified by sex

| Diseases, n(%) | NAFLD (n=1972) | | |  | Other liver diseases (n=1183) | | | No liver diseases (n=6916) | | | |
| --- | --- | --- | --- | --- | --- | --- | --- | --- | --- | --- | --- |
|  | Male  (n=1074) | Female  (n=898) | *P* |  | Male  (n=844) | Female  (n=339) | *P* |  | Male  (n=4248) | Female  (n=2668) | *P* |
| **Liver-related diseases**  diseases | 72(6.7) | 21(2.3) | <0.001 |  | 509(60.3) | 168(49.6) | <0.001 |  | 115(2.7) | 44(1.6) | 0.004 |
| Hepatocellular carcinoma | 37(3.4) | 10(1.1) | <0.001 |  | 258(30.6) | 73(21.5) | <0.001 |  | 75(1.8) | 29(1.1) | 0.024 |
| Decompensated cirrhosis | 13(1.2) | 5(0.6) | 0.234 |  | 165(19.5) | 55(16.2) | 0.156 |  | 15(0.4) | 6(0.2) | 0.345 |
| Hepatic failure | 22(2.0) | 6(0.7) | 0.022 |  | 86(10.2) | 40(11.8) | 0.766 |  | 25(0.6) | 9(0.3) | 0.146 |
| **Extrahepatic neoplasms** | 232(21.6) | 208(23.1) | 0.563 |  | 114(13.5) | 67(19.8) | 0.001 |  | 1240(29.2) | 842(31.6) | 0.037 |
| Colorectal cancer | 70(6.5) | 52(5.8) | 0.489 |  | 30(3.6) | 18(5.3) | 0.168 |  | 207(4.9) | 139(5.2) | 0.531 |
| Lung cancer | 33(3.1) | 26(2.9) | 0.789 |  | 22(2.6) | 11(3.2) | 0.613 |  | 214(5.0) | 146(5.5) | 0.428 |
| Stomach cancer | 25(2.3) | 22(2.4) | 0.928 |  | 17(2.0) | 11(3.2) | 0.189 |  | 169(4.0) | 115(4.3) | 0.498 |
| Hematologic malignancy | 46(4.3) | 48(5.3) | 0.425 |  | 26(3.1) | 18(5.3) | 0.062 |  | 251(5.9) | 171(6.4) | 0.397 |
| Other malignancies | 58(5.4) | 60(6.7) | 0.319 |  | 19(2.3) | 9(2.7) | 0.689 |  | 399(9.4) | 271(10.2) | 0.295 |
| **Cardio- and cerebro- vascular diseases** | 399(37.1) | 314(35.0) | 0.231 |  | 96(11.3) | 40(11.8) | 0.884 |  | 1084(25.5) | 590(22.0) | 0.001 |
| Ischemic heart disease | 249(23.1) | 185(20.6) | 0.225 |  | 33(3.9) | 15(4.4) | 0.746 |  | 660(25.5) | 357(22.1) | 0.001 |
| Cerebrovascular disease | 83(7.7) | 67(7.5) | 0.865 |  | 34(4.0) | 15(4.4) | 0.824 |  | 240(5.6) | 120(4.5) | 0.036 |
| Chronic heart disease | 48(4.5) | 37(4.1) | 0.762 |  | 10(1.2) | 4(1.2) | 0.983 |  | 125(2.9) | 75(2.8) | 0.751 |
| Others | 19(1.8) | 25(2.8) | 0.246 |  | 19(2.3) | 6(1.9) | 0.672 |  | 59(1.4) | 38(1.4) | 0.903 |
| **Infectious diseases** | 134(12.5) | 85(9.5) | 0.034 |  | 29(3.4) | 18(5.3) | 0.115 |  | 408(9.6) | 291(10.8) | 0.109 |
| Pneumonia or sepsis | 83(7.7) | 51(5.8) | 0.120 |  | 19(2.3) | 13(3.8) | 0.144 |  | 257(6.0) | 179(6.7) | 0.272 |
| Aspergillosis | 33(3.1) | 24(2.7) | 0.427 |  | 5(0.6) | 4(1.2) | 0.845 |  | 110(2.6) | 77(2.9) | 0.459 |
| Tuberculosis | 8(0.8) | 4(0.4) | 0.363 |  | 2(0.2) | 1(0.3) | 1.000 |  | 24(0.6) | 17(0.6) | 0.703 |
| Others | 10(0.9) | 6(0.7) | 0.531 |  | 2(0.2) | 1(0.3) | 0.998 |  | 17(0.4) | 18(0.7) | 0.117 |
| **Respiratory diseases** | 45(4.2) | 31(3.5) | 0.332 |  | 18(2.1) | 7(2.1) | 0.936 |  | 361(8.5) | 137(5.1) | <0.001 |
| Chronic obstructive pulmonary diseases | 29(2.7) | 19(2.1) | 0.342 |  | 10(1.2) | 4(1.2) | 0.923 |  | 236(5.6) | 89(3.3) | <0.001 |
| Pulmonary arterial hypertension | 8(0.7) | 7(0.8) | 0.875 |  | 4(0.5) | 1(0.3) | 1.000 |  | 75(1.8) | 29(1.1) | 0.024 |
| Lung fibrosis | 3(0.3) | 2(0.2) | 0.846 |  | 2(0.2) | 1(0.3) | 1.000 |  | 20(0.5) | 7(0.3) | 0.176 |
| Others | 5(0.5) | 3(0.3) | 0.631 |  | 2(0.2) | 1(0.3) | 1.000 |  | 30(0.7) | 12(0.4) | 0.181 |
| **Digestive diseases** | 34(3.2) | 34(3.8) | 0.425 |  | 15(1.8) | 17(5.0) | <0.001 |  | 157(3.7) | 71(2.7) | 0.019 |
| Pancreatitis | 17(1.6) | 19(2.1) | 0.352 |  | 8(0.9) | 9(2.7) | 0.012 |  | 86(2.0) | 38(1.4) | 0.067 |
| Upper gastrointestinal hemorrhage | 13(1.2) | 11(1.2) | 0.952 |  | 5(0.6) | 6(1.8) | 0.136 |  | 58(1.4) | 25(0.9) | 0.111 |
| Others | 4(0.4) | 4(0.4) | 0.896 |  | 2(0.2) | 2(0.6) | 0.717 |  | 13(0.3) | 8(0.3) | 0.964 |
| **Renal diseases** | 76(7.1) | 73(8.1) | 0.422 |  | 39(4.6) | 4(1.2) | 0.001 |  | 263(6.2) | 124(4.7) | 0.008 |
| Chronic kidney diseases | 37(3.4) | 39(4.3) | 0.411 |  | 22(2.6) | 3(0.9) | 0.012 |  | 146(3.4) | 68(2.5) | 0.038 |
| Acute kidney injury | 31(2.9) | 28(3.1) | 0.784 |  | 15(1.8) | 0(0) | 0.034 |  | 94(2.2) | 44(1.6) | 0.103 |
| Others | 8(0.7) | 6(0.7) | 0.824 |  | 2(0.2) | 1(0.3) | 0.742 |  | 23(0.5) | 12(0.4) | 0.601 |
| **Endocrine diseases** | 22(2.0) | 46(5.1) | <0.001 |  | 1(0.1) | 4(1.2) | 0.001 |  | 76(1.8) | 83(3.3) | <0.001 |
| Diabetes mellitus | 15(1.4) | 29(3.2) | 0.004 |  | 0(0) | 2(0.6) | 0.142 |  | 46(1.1) | 51(1.9) | 0.004 |
| Thyrotoxicosis | 5(0.5) | 10(1.1) | 0.083 |  | 1(0.1) | 1(0.3) | 0.514 |  | 20(0.5) | 21(0.8) | 0.095 |
| Others | 2(0.2) | 7(0.8) | 0.052 |  | 0(0) | 1(0.3) | 0.895 |  | 10(0.2) | 11(0.4) | 0.193 |
| **Others** | 60(5.6) | 86(9.6) | <0.001 |  | 23(2.7) | 14(4.1) | 0.165 |  | 544(12.8) | 486(17.9) | <0.001 |

Chi-squared test for the cause of death between male and female in three groups.

Supplementary table 11. Cause of deaths in patients with NAFLD and non-NAFLD with ascribing cryptogenic cirrhosis to Other liver diseases group, stratified by BMI.

| Diseases, n (%) | NAFLD (n=1972) | | |  | Other liver diseases(n=1183) | | |  | No liver diseases  (n=6916) | | |
| --- | --- | --- | --- | --- | --- | --- | --- | --- | --- | --- | --- |
|  | BMI<25  (n=1044) | BMI≥25  (n=928) | *P* |  | BMI<25  (n=910) | BMI≥25  (n=273) | *P* |  | BMI<25  (n=5325) | BMI≥25  (n=1591) | *P* |
| **Liver-related diseases** | 56(5.3) | 37(4.0) | 0.209 |  | 532(51.0) | 145(53.1) | 0.232 |  | 125(2.3) | 34(2.1) | 0.623 |
| Hepatocellular carcinoma | 27(2.6) | 20(2.2) | 0.532 |  | 260(24.9) | 71(26.0) | 0.425 |  | 82(1.5) | 22(1.4) | 0.651 |
| Decompensated cirrhosis | 11(1.1) | 7(0.7) | 0.738 |  | 173(16.6) | 47(17.2) | 0.448 |  | 16(0.3) | 5(0.3) | 1.000 |
| Hepatic failure | 18(1.7) | 10(1.2) | 0.389 |  | 99(9.5) | 27(9.9) | 0.642 |  | 27(0.5) | 7(0.4) | 0.737 |
| **Extrahepatic neoplasms** | 232(22.2) | 208(22.4) | 0.898 |  | 141(13.5) | 40(14.7) | 0.655 |  | 1601(30.1) | 481(30.2) | 0.899 |
| Colorectal cancer | 65(6.2) | 57(6.2) | 0.989 |  | 39(3.7) | 9(3.3) | 0.609 |  | 280(5.3) | 66(4.1) | 0.075 |
| Lung cancer | 32(3.1) | 30(3.2) | 0.944 |  | 26(2.5) | 7(2.6) | 0.964 |  | 270(5.1) | 90(5.7) | 0.356 |
| Stomach cancer | 23(2.2) | 24(2.6) | 0.683 |  | 22(2.1) | 6(2.2) | 0.693 |  | 212(4.0) | 72(4.5) | 0.337 |
| Hematologic malignancy | 51(4.9) | 43(4.6) | 0.936 |  | 33(3.1) | 11(4.0) | 0.867 |  | 315(5.9) | 107(6.7) | 0.236 |
| Other malignancies | 61(5.8) | 54(5.8) | 0.818 |  | 21(2.0) | 7(2.6) | 0.893 |  | 524(9.8) | 146(9.2) | 0.432 |
| **Cardio- and cerebrovascular diseases** | 321(30.7) | 392(42.2) | <0.001 |  | 91(8.7) | 45(16.5) | 0.001 |  | 1208(22.7) | 466(29.3) | <0.001 |
| Ischemic heart disease | 196(18.8) | 238(25.6) | <0.001 |  | 32(3.1) | 16(5.9) | 0.225 |  | 734(13.8) | 283(17.8) | <0.001 |
| Cerebrovascular disease | 67(6.4) | 83(8.9) | 0.049 |  | 32(3.1) | 16(5.9) | 0.078 |  | 260(4.9) | 100(6.3) | 0.027 |
| Chronic heart disease | 36(3.4) | 49(5.4) | 0.025 |  | 9(0.9) | 6(2.2) | 0.269 |  | 144(2.7) | 56(3.5) | 0.088 |
| Others | 22(2.1) | 22(2.4) | 0.783 |  | 19(1.8) | 6(2.2) | 0.629 |  | 70(1.3) | 27(1.7) | 0.255 |
| **Infectious diseases** | 141(13.5) | 78(8.4) | <0.001 |  | 38(3.6) | 9(3.3) | 0.452 |  | 562(10.6) | 137(8.6) | 0.024 |
| Pneumonia or sepsis | 88(8.4) | 47(5.1) | 0.001 |  | 27(2.6) | 4(1.5) | 0.413 |  | 350(6.6) | 86(5.4) | 0.093 |
| Aspergillosis | 36(3.4) | 21(2.2) | 0.052 |  | 7(0.7) | 2(0.7) | 0.876 |  | 150(2.8) | 27(1.7) | 0.013 |
| Tuberculosis | 8(0.8) | 3(0.3) | 0.452 |  | 2(0.2） | 2(0.7) | 1.000 |  | 33(0.6) | 8(0.5) | 0.594 |
| Others | 9(0.9) | 7(0.8) | 0.632 |  | 2(0.2） | 1(0.4) | 1.000 |  | 29(0.5) | 6(0.4) | 0.409 |
| **Respiratory diseases** | 43(4.1) | 33(3.6) | 0.532 |  | 19(1.8) | 6(2.2) | 0.948 |  | 403(7.6) | 95(6.0) | 0.031 |
| Chronic obstructive pulmonary diseases | 27(2.6) | 22(2.4) | 0.523 |  | 10(1.0) | 1(0.4) | 0.890 |  | 263(4.9) | 62(3.9) | 0.085 |
| Pulmonary arterial hypertension | 8(0.8) | 7(0.8) | 0.782 |  | 4(0.4) | 2(0.7) | 0.930 |  | 84(1.6) | 20(1.3) | 0.357 |
| Lung fibrosis | 2(0.2) | 2(0.2) | 0.922 |  | 2(0.2） | 2(0.7) | 0.95 |  | 22(0.4) | 5(0.3) | 0.579 |
| Others | 6(0.6) | 2(0.5) | 0.936 |  | 3(0.3) | 1(0.4) | 0.7920 |  | 34(0.6) | 8(0.5) | 0.541 |
| **Digestive diseases** | 38(3.6) | 30(3.2) | 0.647 |  | 24(2.3) | 8(2.9) | 0.956 |  | 185(3.5) | 43(2.7) | 0.130 |
| Pancreatitis | 20(2.0) | 17(1.8) | 0.762 |  | 13(1.2) | 3(1.5) | 1.000 |  | 100(1.9) | 24(1.5) | 0.330 |
| Upper gastrointestinal hemorrhage | 13(1.2) | 10(1.1) | 0.926 |  | 8(0.8) | 4(1.5) | 1.000 |  | 68(1.3) | 15(0.9) | 0.283 |
| Others | 5(0.5) | 3(0.3) | 0.831 |  | 3(0.3) | 1(0.4) | 1.000 |  | 17(0.3) | 4(0.3) | 0.666 |
| **Renal diseases** | 92(8.8) | 57(6.1) | 0.032 |  | 33(3.2) | 10(3.7) | 0.856 |  | 305(5.7) | 82(5.2) | 0.382 |
| [Chronic](D:/Program%20Files%20(x86)/Youdao/Dict/8.5.3.0/resultui/html/index.html#/javascript:;) [kidney](D:/Program%20Files%20(x86)/Youdao/Dict/8.5.3.0/resultui/html/index.html#/javascript:;) [diseases](D:/Program%20Files%20(x86)/Youdao/Dict/8.5.3.0/resultui/html/index.html#/javascript:;) | 48(4.6) | 29(3.1) | 0.110 |  | 19(1.8) | 5(1.8) | 0.934 |  | 169(3.2) | 45(2.8) | 0.485 |
| Acute kidney injury | 36(3.4) | 22(2.4) | 0.234 |  | 12(1.1) | 4(1.5) | 0.785 |  | 109(2.0) | 29(1.8) | 0.575 |
| Others | 8(0.8) | 6(0.6) | 0.763 |  | 2(0.2） | 1(0.4) | 1.000 |  | 27(0.5) | 8(0.5) | 0.983 |
| **Endocrine diseases** | 30(2.9) | 38(4.1) | 0.115 |  | 2(0.2) | 3(1.1) | 0.223 |  | 111(2.1) | 48(3.0) | 0.029 |
| [Diabetes](D:/Program%20Files%20(x86)/Youdao/Dict/8.5.3.0/resultui/html/index.html#/javascript:;) [mellitus](D:/Program%20Files%20(x86)/Youdao/Dict/8.5.3.0/resultui/html/index.html#/javascript:;) | 19(1.8) | 26(2.8) | 0.295 |  | 2(0.2) | 1(0.4) | 0.959 |  | 68(1.3) | 29(1.8) | 0.104 |
| Thyrotoxicosis | 5(0.5) | 9(1.0) | 0.442 |  | 0(0) | 2(0.7) | 0.542 |  | 28(0.5) | 13(0.8) | 0.184 |
| Others | 6(0.6) | 3(0.3) | 0.605 |  | 0(0) | 0(0) | - |  | 15(0.3) | 6(0.4) | 0.544 |
| **Others** | 91(8.7) | 55(5.9) | 0.034 |  | 30(2.9) | 7 (2.6) | 0.546 |  | 825(15.5) | 205(12.9) | 0.010 |

Abbreviations: BMI, body mass index.

Chi-squared test for the cause of death between BMI<25 and BMI≥25 in three groups

Supplementary table 12. Cause of deaths in patients with NAFLD without including cryptogenic cirrhosis, stratified by FIB-4.

| Diseases, n(%) | FIB-4 < 1.3  (n=1607) | FIB-4 ≥ 1.3  (n=365) | *P* |
| --- | --- | --- | --- |
| **Liver-related diseases**  diseases | 61(3.8) | 32(8.8) | <0.001 |
| Hepatocellular carcinoma | 29(1.8) | 18(4.9) | <0.001 |
| Decompensated cirrhosis | 13(0.8) | 5(1.4) | 0.125 |
| Hepatic failure | 19(1.2) | 9(2.5) | 0.049 |
| **Extrahepatic neoplasms** | 344(21.7) | 96(26.3) | 0.049 |
| Colorectal cancer | 89(5.6) | 33(9.0) | 0.032 |
| Lung cancer | 47 (3.0) | 15(4.1) | 0.652 |
| Stomach cancer | 34(2.2) | 13(3.6) | 0.764 |
| Hematologic malignancy | 79(5.0) | 15(4.1) | 0.582 |
| Other malignancies | 95(6.0) | 20(5.5) | 0.367 |
| **Cardio- and cerebro- vascular diseases** | 554(34.5) | 159(43.6) | <0.001 |
| Ischemic heart disease | 334(20.8) | 100(27.4) | <0.001 |
| Cerebrovascular disease | 119(7.4) | 31(8.5) | 0.512 |
| Chronic heart disease | 69(4.3) | 16(4.4) | 0.804 |
| Others | 32(2.0) | 12(3.3) | 0.367 |
| **Infectious diseases** | 191(11.9) | 28(7.7) | 0.030 |
| Pneumonia or sepsis | 117(7.3) | 18(4.9) | 0.046 |
| Aspergillosis | 50(3.1) | 7(1.9) | 0.215 |
| Tuberculosis | 10(0.6) | 1(0.3) | 1.000 |
| Others | 14(0.9) | 2(0.5) | 0.665 |
| **Respiratory diseases** | 69(4.3) | 7(1.9) | 0.065 |
| Chronic obstructive pulmonary diseases | 44(2.7) | 5(1.4) | 0.125 |
| Pulmonary arterial hypertension | 14(0.9) | 1(0.3) | 0.525 |
| Lung fibrosis | 4(0.2) | 0(0) | 1.000 |
| Others | 7(0.4) | 1(0.3) | 1.000 |
| **Digestive diseases** | 61(3.8) | 7(1.9) | 0.320 |
| Pancreatitis | 33(2.1) | 4(1.1) | 0.202 |
| Upper gastrointestinal hemorrhage | 21(1.3) | 3(0.8) | 0.347 |
| Others | 7(0.4) | 1(0.3) | 1.000 |
| **Renal diseases** | 129(8.0) | 20(5.5) | 0.065 |
| Chronic kidney diseases | 67(4.2) | 10(2.7) | 0.126 |
| Acute kidney injury | 50(3.1) | 8(2.1) | 0.315 |
| Others | 12(0.7) | 2(0.5) | 0.563 |
| **Endocrine diseases** | 59(3.7) | 9(2.5) | 0.236 |
| Diabetes mellitus | 39(2.4) | 6(1.6) | 0.354 |
| Thyrotoxicosis | 11(0.7) | 3(0.8) | 0.982 |
| Others | 9(0.6) | 0(0) | 1.000 |
| **Others** | 139(8.3) | 7(1.9) | <0.001 |

Abbreviations: FIB-4, fibrosis index based on the 4 factor

Chi-squared test for the cause of death between FIB-4 < 1.3 and FIB-4 ≥ 1.3 in NAFLD group.
